# FBLN2 is associated with basal cell markers Krt14 and ITGB1 in mouse mammary epithelial cells and has a preferential expression in molecular subtypes of human breast cancer

**DOI:** 10.1007/s10549-024-07447-y

**Published:** 2024-08-07

**Authors:** Amr Ahmed WalyEldeen, Salwa Sabet, Shady E. Anis, Torsten Stein, Ayman M. Ibrahim

**Affiliations:** 1 Department of Zoology, Faculty of Science, Cairo University, Giza, 12613 Egypt; 2 Department of Pathology, Faculty of Medicine, Cairo University, Cairo, 11562 Egypt; 3 Institute of Veterinary Biochemistry, Freie Universität Berlin, 14163 Berlin, Germany; 4 Institute of Cancer Sciences, College of MVLS, University of Glasgow, Glasgow, G12 8QQ UK; 5 Aswan Heart Centre, Magdi Yacoub Heart Foundation, Aswan, Egypt

**Keywords:** Fibulin-2, ECM, Basal, Breast cancer, Molecular subtypes

## Abstract

**Background:**

Fibulin-2 (FBLN2) is a secreted extracellular matrix (ECM) glycoprotein and has been identified in the mouse mammary gland, in cap cells of terminal end buds (TEBs) during puberty, and around myoepithelial cells during early pregnancy. It is required for basement membrane (BM) integrity in mammary epithelium, and its loss has been associated with human breast cancer invasion. Herein, we attempted to confirm the relevance of FBLN2 to myoepithelial phenotype in mammary epithelium and to assess its expression in molecular subtypes of human breast cancer.

**Methods:**

The relationship between FBLN2 expression and epithelial markers was investigated in pubertal mouse mammary glands and the EpH4 mouse mammary epithelial cell line using immunohistochemistry, immunocytochemistry, and immunoblotting. Human breast cancer mRNA data from the METABRIC and TCGA datasets from Bioportal were analyzed to assess the association of *Fbln2* expression with epithelial markers, and with molecular subtypes. Survival curves were generated using data from the METABRIC dataset and the KM databases.

**Results:**

FBLN2 knockdown in mouse mammary epithelial cells was associated with a reduction in KRT14 and an increase in KRT18. Further, TGFβ3 treatment resulted in the upregulation of FBLN2 in vitro. Meta-analyses of human breast cancer datasets from Bioportal showed a higher expression of *Fbln2* mRNA in claudin-low, LumA, and normal-like breast cancers compared to LumB, Her2 +, and Basal-like subgroups. *Fbln2* mRNA levels were positively associated with mesenchymal markers, myoepithelial markers, and markers of epithelial–mesenchymal transition. Higher expression of *Fbln2* mRNA was associated with better prognosis in less advanced breast cancer and this pattern was reversed in more advanced lesions.

**Conclusion:**

With further validation, these observations may offer a molecular prognostic tool for human breast cancer for more personalized therapeutic approaches.

**Supplementary Information:**

The online version contains supplementary material available at 10.1007/s10549-024-07447-y.

## Introduction

Breast cancer is the most prevalent type of cancer and the leading cause of mortality among women worldwide [1, 2]. The complexity and heterogeneity of breast cancer make it challenging to accurately describe the causing factors that simultaneously promote cancer cells, and disease progression and eventually predict patient’s prognosis [3]. While almost all breast cancers arise in the breast’s ductal lobular units, cancer cells are enclosed within a complex microenvironment of stromal cells, including immune cells, fibroblasts, and adipocytes, all embedded in an ECM that is produced and interact with stromal cells [4–7].

In physiological conditions, ECM is required to maintain tissue homeostasis in conjunction with various cell components in response to cell–cell and cell–ECM interaction [8]. ECM remodeling associated with cancer development occurs via the breakup of old ECM proteins using various proteases, and the formation of new proteins including fibrillar collagens, BM proteins, and other glycoproteins that make up the ECM niche [9]. It is therefore essential to dissect the ECM in the breast cancer microenvironment and the mechanisms of tissue remodeling that govern tumor development in different cancer subtypes.

FBLN2 is a secreted ECM glycoprotein that has been associated with epithelial–mesenchymal (EMT) transition during endocardium formation, and tissue remodeling during myocardial fibrosis [10], in addition to elastin and collagen fibers stability during vascular developmental stages, and aortopathies [11]. In the mouse mammary gland, FBLN2 has been specifically detected at the myoepithelial cells of TEBs during puberty and myoepithelial cells during early pregnancy when the duct-associated ECM is remodeled [12–14]. FBLN2 can bind to multiple ECM-related proteins, including integrins [15], laminin [16, 17], fibronectin [18], aggrecan, versican [19], nidogen [20], perlecan [21, 22], and tropoelastin [23]. We have recently reported the importance of FBLN2 in the formation of a stable BM during mammary gland morphogenesis and the gradual loss of its expression in areas of tumor invasion in human breast cancer [14].

In this study, we aimed to confirm the association of FBLN2 with myoepithelial phenotype in mammary epithelium and further investigate the expression of FBLN2 in human breast cancer subtypes in relevance to patients’ outcomes.

## Materials and methods

### Animal husbandry and mammary glands tissue processing

All animals were kept according to the UK Home Office guidelines and the project was approved by the University of Glasgow ethics committee and in accordance with ARRIVE guidelines (https://arriveguidelines.org). 6-week-old C57BL/6 mice were purchased from Harlan Laboratories UK. Inguinal mammary glands from 7 weeks old were excised, fixed in 10% Neutral buffered formalin, and then processed to produce formalin-fixed paraffin-embedded (FFPE) blocks.

### Immunofluorescence

Three to 4 μm sections were routinely cut from FFPE mammary glands tissues and staining was performed as previously described [14]. Briefly, antigen retrieval was performed using 1 mM EDTA buffer (pH 8) under high pressure and all incubations were performed at RT using a humidity chamber. Sections were blocked with Image-iT™ FX Signal Enhancer (Invitrogen), and then incubated with primary antibody for 30 min (anti-FBLN2 1:10,000 [24], anti-Integrin β1(1:200) [Abcam, Ab52971], and anti-integrin α3 (1:300) [Mike DiPersio lab]. Alexa Fluor conjugated secondary antibodies (Alexa Fluor 488; Alexa Fluor 594, Invitrogen) were used at 1:1000 dilution and incubated for 20 min in dark. Tissue sections were mounted using ProLong® Gold antifade reagent with DAPI (Invitrogen) and then imaged using a Leica TCS SP2 confocal microscope with 63 × oil immersion lens.

### Cell culture and transduction

EpH4 (normal mouse mammary epithelial cells) and Mouse embryonic fibroblasts (MEFs) were maintained in Dulbecco’s Modified Eagle Medium (DMEM) supplemented with 10% FBS, 2 mM l-glutamine, and 1% penicillin/streptomycin. Cells were cultivated in a humidified atmosphere with 5% CO_2_ at 37 °C. For TGFβ3 treatment experiments, cells were plated in 6-well plates at equal density per well, incubated till 80–90% confluence and then media were changed to serum-free medium with ascending concentrations of TGFβ3; 0.5, 2, 5, 25, and 50 ng/ml in addition to a control untreated well. Cells were incubated for 24 h, and then were harvested for protein and RNA isolation. For the knockdown (KD) experiment, lentivirus production for EpH4 cell transduction was performed as described before [14]. Briefly, HEK-293 cells were transfected using X-tremeGENE 9 (Roche Diagnostics Ltd., Burgess Hill, UK), viral vector components (Thermo Fisher), and 3 μg of Lentiviral shRNA three pLKO1-Puro vectors which target all FBLN2 mouse variants regulated by human U6 promotor (Clones: TRCN0000109479, TRCN0000109478, and TRCN0000109476 (Thermo Fisher) or scrambled control (Scr) shRNA pLKO1-puro control vector (Thermo Fisher). Cells were treated with the resulting transfection solution overnight, followed by 24 h incubation with fresh medium, after which virus-containing material was collected and filtered using syringe-driven filters (Millipore Ltd., Livingstone, UK). Filtered virus was then added to EpH4 cells in a 6-well plate and incubated for 4 h at 37 °C in an incubator containing 5% CO_2_, cells were covered with DMEM/10% FBS media supplemented with 8 μg/mL polybrene (Sigma) and cultured for an additional 24 h. Media were changed to a freshly prepared medium that included 3 μg/ml of puromycin (Sigma) to select for successfully transduced cells. FBLN2 KD was confirmed using RNA and protein analyses as previously reported [14].

### Immunoblotting

Protein isolation and western blot were performed as previously described [14]. Briefly, equal concentrations of denatured proteins, isolated with RIPA buffer, were separated in 4–12% BIS–Tris-HCl gels using Novex NuPage™ (Thermo Fisher) with 1xNuPage MES SDS buffer. A Novex XCell IITM Blot module (Thermo Fisher) transferred proteins onto Whatman® Protran® Nitrocellulose Transfer Membrane (0.2 μm) (GE Healthcare Biosciences). After blocking, blot was incubated for 2 h at RT with primary antibody (Krt18 1:100 [Proteintech, 08-708-1], Krt14 1:100 [Proteintech, 10-43-1], and β-actin 1:500 [Sant Cruz Biotechnologies, Sc-1615]). Blot was washed thrice and then incubated for 1 h at RT with horseradish peroxidase (HRP)-labeled secondary antibody (Thermo Fisher) in blocking buffer. Blot was incubated with ECL Western blotting detection reagent (Thermo Fisher), and signal was detected using Fujifilm UK Ltd.'s Intelligent Dark Box LAS-3000 (LAS-3000 v.2.2, Fujifilm).

#### RNA isolation and quantitative RT-qPCR

RNA was extracted and cDNA was produced using 500 ng of RNA as previously described [14]. qPCR reactions were carried out using a LightCycler® 480 Instrument (Roche). Each reaction contained 0.25 μM in 1 μl of probe, 7.2 μM of both forward and reverse primers in 1 μl, 10 μl of 2 × LightCycler® 480 TaqMan Master Mix (Roche), 5 μl of diluted cDNA and dH_2_O to a final reaction volume of 20 μl. Primers (Sigma) used: *Fbln2*: Fwd 5′-tgttgttggggacacagcta-3′, Rev 5′- ccatcaaacactcgtcttggt-3′, Probe (#22), *βactin*: Fwd 5′-aaggccaaccgtgaaaagat-3′, Rev 5′-gtggtacgaccagaggcatac-3′, probe (#56). Relative mRNA expression was calculated by the 2^−ΔΔCt^ method.

### Flow cytometry

FBLN2 KD and Scr cells were trypsinised and resuspended in PBS at 1 × 10^6^ cells/ml. Cells were then stained with anti-CD29 (710 +, BD biosciences, 550,531) and anti-CD24 (PE + , BD biosciences, 550,531) antibodies in L15/10% FCS for 30 min with frequent shaking, and then washed thrice in L15/10% FCS and resuspended in 4',6-diamidino-2-phenylindole dihydrochloride (DAPI) for dead cells staining. With the exclusion of dead cells, debris and clumped cells, each line was analyzed based on forward- and side-scatter, using BD FACSARIA II (BD) provided with FACSDiva Version 6.1.3 software. 100,000 cells were tested per experiment.

### Human subjects and subtypes assessment

Histological sections from 65 patients’ tissues and stained with FBLN2 were retrospectively analyzed upon categorization to breast cancer subtypes. The study received an ethical approval from Cairo University ethics committee (IRB: N-8-2017). Hormonal receptor and growth factor receptors expression data (Erα, PR, and Her2) were acquired along with other clinical and pathological characteristics of the patient cohort, such as tumor grade, lymph node (LN) involvement, and lymph-vascular invasion. Patients’ clinical and histopathological characteristics were previously reported [14]. Patients were categorized as luminal A (LumA), luminal B (LumB), Her2 +, and basal-like, based on hormone receptor status, Her2 status, and Ki67 expression [25–27]. 37 patients had complete data for this categorization as follows: lumA (*n* = 8), LumB (*n* = 13), Her2 + (*n* = 4), and basal-like (*n* = 9). In addition, patients were also sub-grouped according to their LN status. Statistical analysis of FBLN2 expression among subtypes was performed using a Kruskal–Wallis test, while a Mann–Whitney test was performed for Erα, PR, Her2, LN status, and KI67.

### Publicly available data and statistical analyses

Human breast cancer mRNA data were extracted from the two largest online datasets METABRIC (2509 breast cancer patients) and TCGA (825 breast cancer patients) through Bioportal [https://www.cbioportal.org/] [28–31], as previously described [32]. In addition, we extracted data for *Fbln2* mRNA expression from whole transcriptome data [RNA-seq] performed in breast cancer cell lines from two datasets: the Cancer Cell Encyclopedia through Bioportal [https://www.cbioportal.org/] (47 breast cancer cell line) [33, 34], and the Human Protein Atlas (50 breast cancer Cell line) [https://www.proteinatlas.org/] [35]. We further extracted mRNA expression data from the Kaplan–Meier (KM) database that comprises gene microarray data and patient survival rates from Gene Expression Omnibus (Affymetrix HGU133A and HGU133 + 2 microarrays) (https://kmplot.com/, 4929 breast cancer patients) [36]. Correlation Matrix between *Fbln2* versus luminal markers (*Krt18, Krt19, Ddr1, Krt8*), epithelial markers (*Itgb1, Krt7, Krt5, Krt17, Krt14*), and other *Fblns* (*Fbln1* and *Fbln5*), using data from the METABRIC database and the KM database. Two variables’ correlation was assessed using Pearson’s correlation. mRNA expression data analysis based on molecular subtypes was performed using data from the METABRIC and TCGA databases. One-way analysis of variance (ANOVA) was performed to compare more than two groups, and Tukey’s post hoc test was performed to test the difference between each two subgroups. Data were presented as mean ± SEM, and all tests were considered significant at a *p* value < 0.05. GraphPad Prism 8 was used to create the graphs.

Survival curves were generated using data from the METABRIC dataset and the KM databases [36]. For METABRIC, data were mined, and overall survival (OS) statuses of patients with different subtypes and the corresponding *Fbln2* mRNA level per patient were downloaded and grouped as high or low, based on their median expression as a cutoff. Survival graphs were then plotted using survival module in GraphPad, Prism8. In KM database, patients were divided using an auto-selection feature based on median and quartile expression levels of *Fbln2* (valid Affy ID: 203886_s_at). OS and relapse-free survival (RFS) were tested without further criteria filtering. We analyzed the OS, RFS, and distant metastasis-free survival (DMFS) with and without patient filtering per molecular subtypes (LumA, LumB, Her2 + , Basal), ER status (positive and negative), PR status (positive and negative), Her2 status (positive and negative), grades (grade 1, 2, and 3), and lymph node status (positive and negative). According to the Chi-squared test and Log-rank, a *p*-value < 0.05 was considered statistically significant.

## Results

### FBLN2 Co-localizes with ITGβ1 and ITGα3 in the TEB Cap Calls

**Fig. 1:**
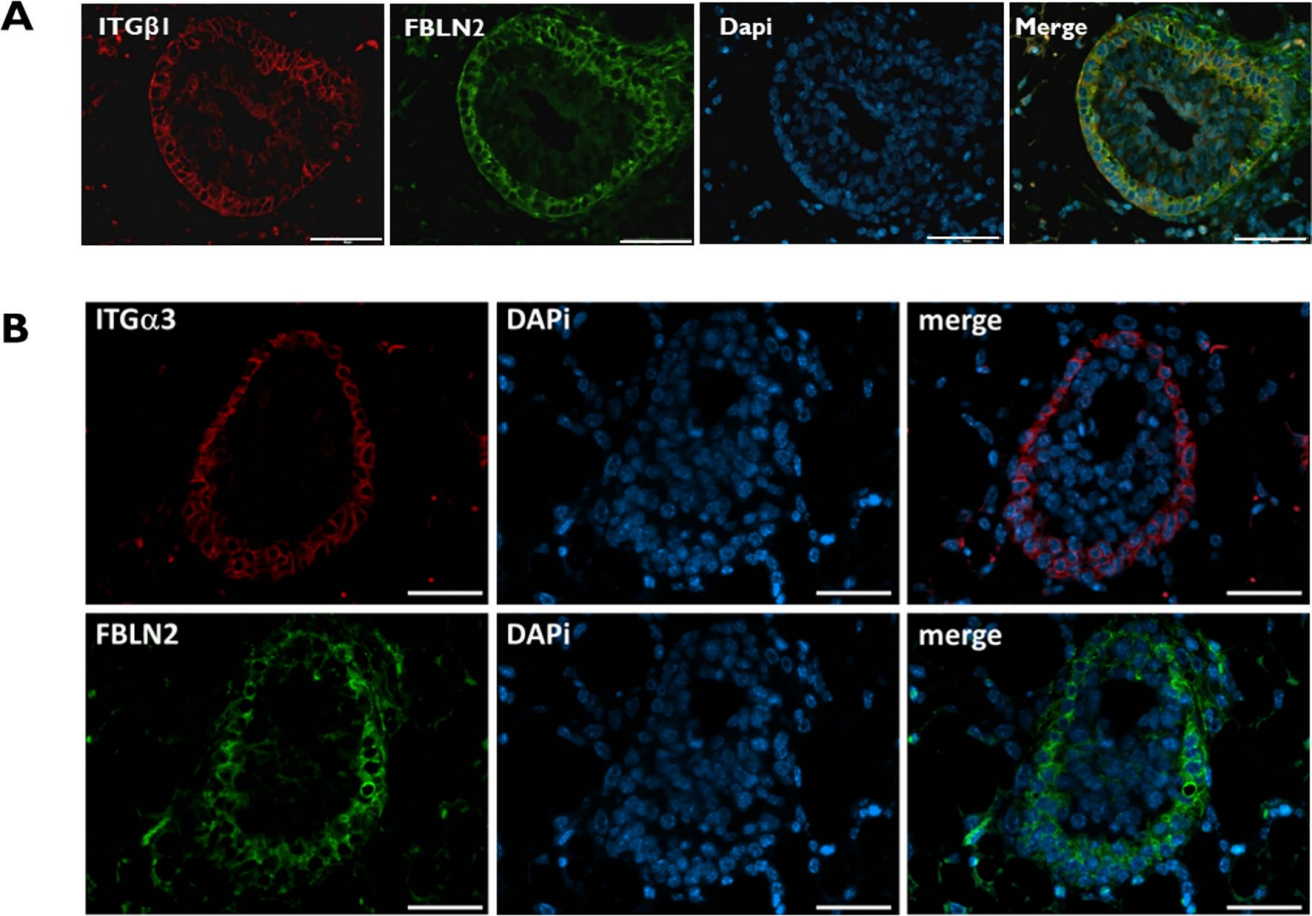
FBLN2 is co-localized with ITGβ1 and ITGα3 in the terminal end buds of pubertal mouse mammary gland in mice. **a** Double staining of ITGβ1 and FBLN2 in TEBs of the mouse pubertal mammary gland showing co-localization of both markers at the cap cells, and not in the luminal cells (*n* = 3). Bars represent 50 μm.** b** Immunofluorescence staining of ITGα3 and FBLN2 in consecutive sections of TEBs of pubertal mouse mammary gland showing co-localization of both markers at the cap cells, and not in the luminal cells (*n* = 3)

We have previously reported that FBLN2 is preferentially expressed at the terminal end buds (TEBs) of the pubertal mouse mammary gland and not in the ductal epithelium [14]. Using immunofluorescence, we here showed that ITGβ1 and ITGα3, markers of myoepithelial cells [37–40] exhibit similar patterns of expression to FBLN2 (Supplementary Fig. 1). To further assess their relative co-localization in the TEBs, we performed a co-immunofluorescence staining which showed that FBLN2 is exclusively expressed at the cap cells of TEBs, and not in luminal cells, co-localizing with ITGβ1 and ITGα3 (Fig. 1a, b and supplementary Fig. 2).

### TGFβ3 stimulates FBLN2 overexpression in mouse mammary epithelial cells

**Fig. 2:**
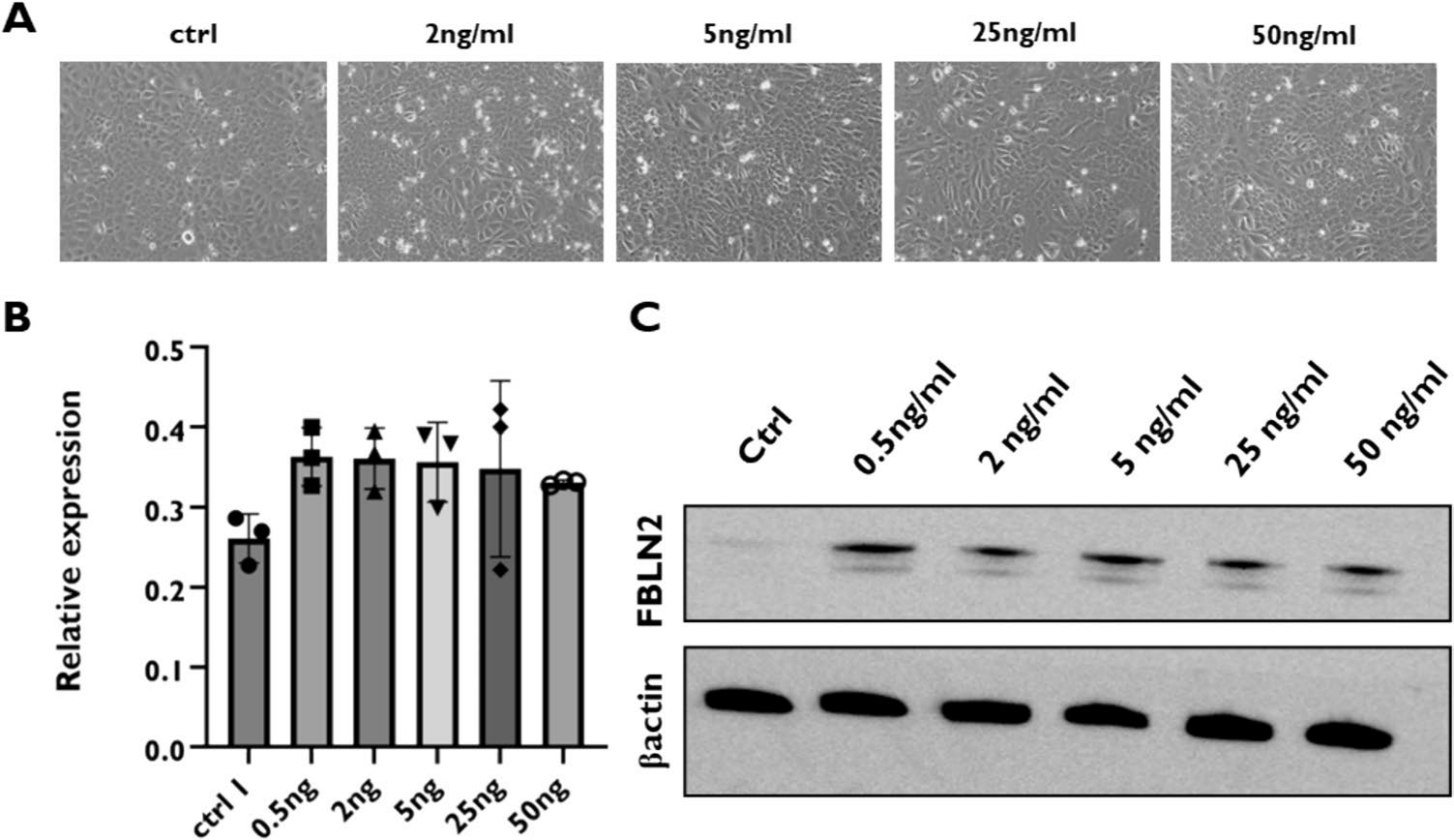
Effect of TGFB3 treatment on FBLN2 expression in EpH4 cells. **a** Phase contrast microscopy of cells showing the transformation to a spindle-shaped morphology, reminiscent of myoepithelial cells, upon treatment of cells with TGFβ3. **b** Relative mRNA expression of *Fbln2* upon treatment of EpH4 cells with different concentrations of TGFβ3 showing the upregulation of *Fbln2* in a dose-independent manner. Error bars represent the standard error of three biological replicates. **c** Immunoblotting of FBLN2 expression upon treatment of EpH4 cells with ascending concentrations of TGFβ3, confirming the upregulation of FBLN2 after TGFβ3 treatment, relative to βactin expression (*n* = 3)

As TGFβ3 is also mainly expressed in the cap cells of TEBs [41] and drives BM protein expression, we asked whether TGFβ3 treatment will stimulate FBLN2 expression in mammary epithelial cells. TGFβ3 treatment of the morphologically normal mouse mammary cell line induced the transformation to a spindle-shaped morphology, a characteristic of myoepithelial cells, (Fig. 2a). It also induced the expression of FBLN2 in a dose-independent manner, evident both on mRNA and protein levels (Fig. 2b and c); however, it was statistically insignificant. Further, TGFβ3 treatment of spontaneously immortalized mouse embryonic fibroblasts (MEFs) resulted in an equally dose-independent upregulation of the FBLN2 protein (Supplementary Fig. 3).

### FBLN2 KD drives a luminal phenotype in mouse mammary cells

**Fig. 3:**
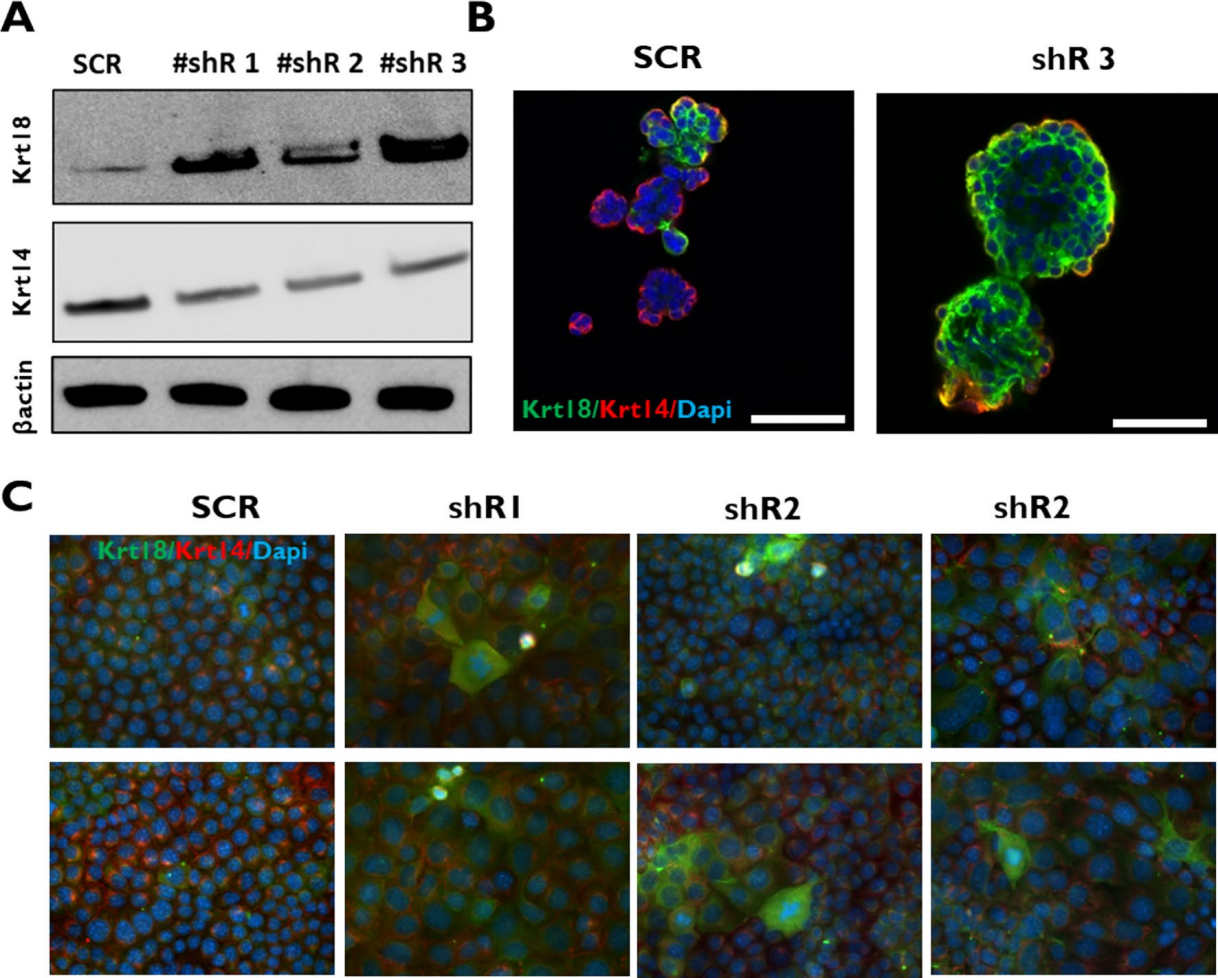
FBLN2 KD correlates with a reduction in the basal cell marker Krt14 and an increase in luminal cell marker Krt18. **a** Immunoblotting analysis of Krt18 and Krt14 in control and FBLN2 KD cells using three shr1 (70%), 2 (28%), and 3 (18%) FBLN2 expression cells. Expression was relative to βactin (*n* = 3). **b** Immunofluorescence staining of Krt18 and Krt14 in Matrigel-embedded control and FBLN2 KD cells [70% reduction in FBLN2 expression cells]. Scale bars are 50 µm (*n* = 3). **c** Immunofluorescence staining of Krt18 and Krt14 in monolayered control and FBLN2 KD cells (*n* = 3). Scale bars are 50 µm

To further examine the association of FBLN2 to basal phenotype in mammary cells, we assessed the expression of the myoepithelial/basal marker Krt14 [42, 43], and the luminal marker Krt18 [42, 44] in EpH4 cells in which FBLN2 was KD. Immunoblotting analysis of EpH4 cell lysates with different levels of FBLN2 KD (80, 28, and 18%) [14] showed a reduced expression of KRT14 and an increased expression of KRT18 upon FBLN2 KD (Fig. 3a). Flow cytometry analysis of one biological replicate confirmed this shift toward luminal phenotype upon FBLN2 KD (Supplementary Fig. 4). Immunofluorescence staining using monolayered and Matrigel-embedded cells also showed an increased expression of KRT18; however, the levels of expression among cells appeared heterogeneous, where cells with an increased expression of KRT18 being larger in size in monolayer cultures (Fig. 2c and 3b).

### Fbln2 mRNA is preferentially correlated to tuman mammary basal markers

**Fig. 4:**
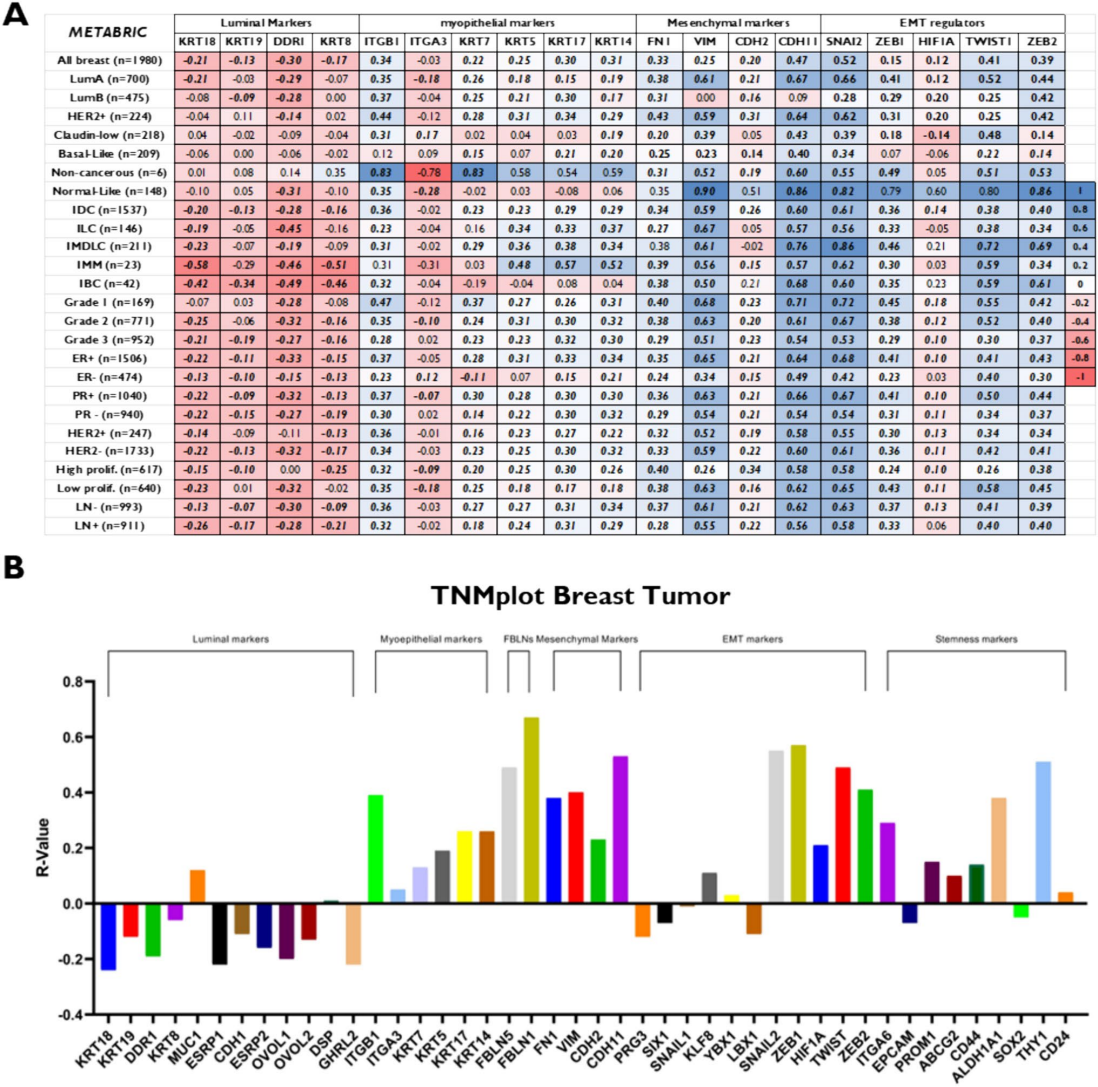
*Fbln2* mRNA expression positively correlates with basal and mesenchymal markers in human breast cancer.** a** Correlation matrix of data retrieved from online datasets (METABRIC) for *Fbln2* against luminal, myoepithelial, EMT, and mesenchymal markers. Numbers refer to the R coefficient of Pearson correlation between *Flbn2* and each marker.** b** Correlation matrix generated by TNMplot.com for *Fbln2* with luminal, myoepithelial cell markers, EMT, mesenchymal, stemness, and other *Fblns* in breast tumor. ER + Her2 − HP: ER + Her2 − High proliferation, ER + Her2 − LP: ER + Her2 − Low Proliferation, IDC: Invasive Ductal Carcinoma, ILC: Invasive Lobular Carcinoma, IMM: Invasive Mixed Mucinous, IMDLC: Invasive Mixed Ductal and Lobular Carcinoma, and IBC: Invasive Breast Carcinoma

To investigate the preferential expression of FBLN2 in basal cells in human breast cancer, we retrieved online datasets from the Bioportal [28–30] (METABRIC dataset) and assessed the correlation between mRNA levels of *Fbln2* and the available data on luminal, myoepithelial, EMT, stemness, and mesenchymal markers. Generally, METABRIC dataset analysis showed a negative correlation of *Fbln2* with luminal markers (*Krt18*, *Krt19*, *Ddr1*, and *Krt8*) [37–40], and a positive correlation with myoepithelial cell markers (*Krt14*, *Itgb1*, *ITtga3, Krt7, Krt5, Krt17*, and *Krt14*) [37–40] and mesenchymal markers (*Fn, Vim, Cdh2,* and *Cdh11*) [45] in the majority of the molecular and clinical subtypes of breast cancer patients [including grouping based on hormonal receptors, LN status, tumor grade, growth factor receptor status, and tumor proliferation] (Fig. 4a). Markers of EMT (*Sna12, Zeb1, Twist1, Zeb2,*) [45] and markers of stemness (*Itga6, Epcam, Prom1,Abcg2, Cd44, Aldh1a1, Sox2, Thy1,* and *Cd24*) [46] showed different patterns of correlations with *Fbln2* (Fig. 4a and Supplementary Fig. 5a). Of note, *Fbln2* had significant positive correlations with *Fbln1* and *Fbln5* in all breast cancer subtypes (Fig. 4 and Supplementary Fig. 5). *P* values, and number of patients per group are summarized in Supplemental Table 1.

Further, analysis of the retrieved dataset from TNMplot tool in KM database showed the same pattern of correlation with *Fbln2* in breast cancer group (*n* = 7569) (Fig. 4b); however, myoepithelial markers showed a negative correlation with *Fbln2*, except for *Itgb1*, in the morphologically normal breast group (*n* = 242) (Supplementary Fig. 5b).

In addition, the relation of fbln2 to basal/mesenchymal and stemness phenotype within mammary gland was validated using the data extracted from Girardi’s datasets [47] (Supplementary Fig. 6).

### Fbln2 mRNA has a distinct expression in different subtypes of human breast cancer

**Fig. 5:**
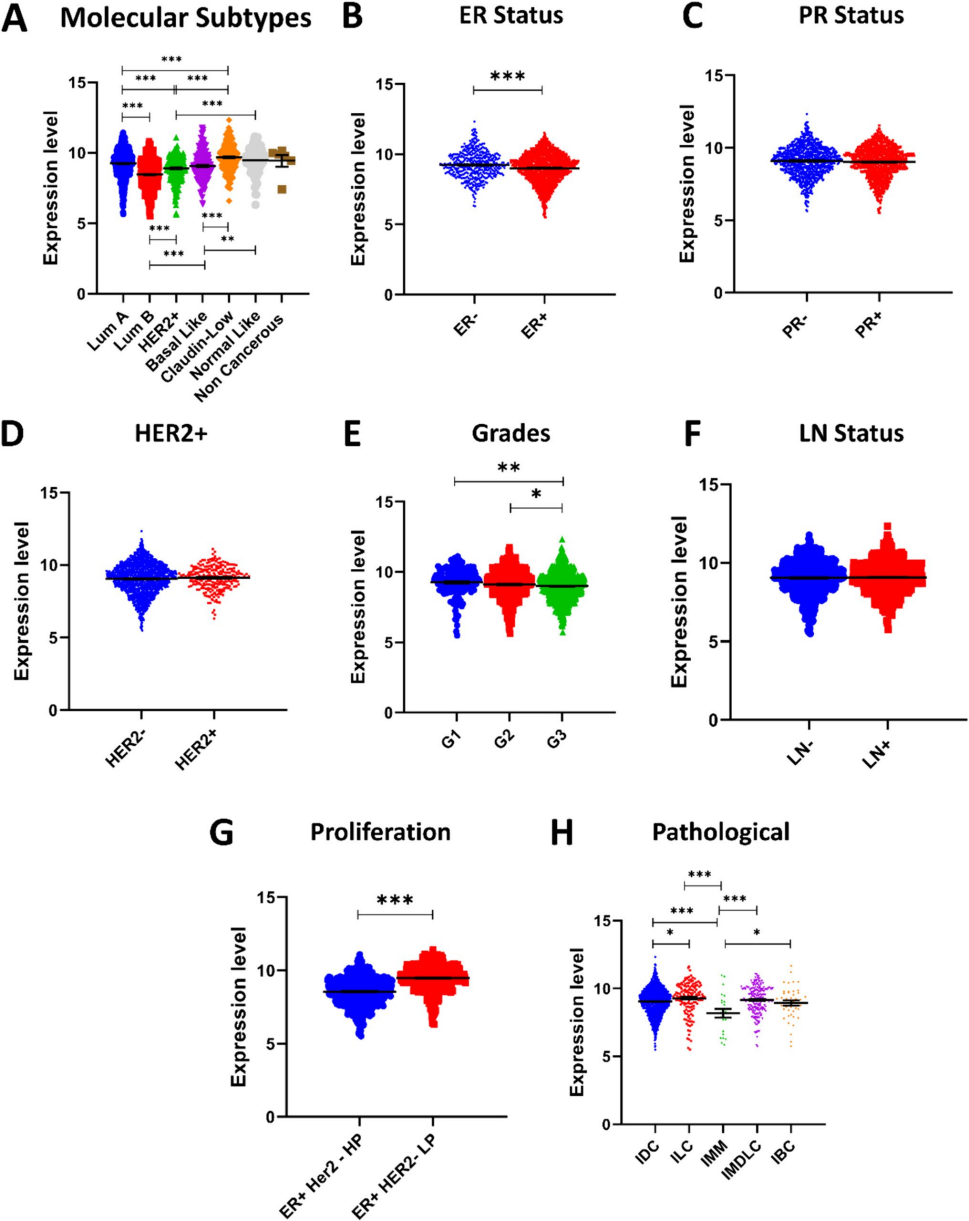
Preferential expression of FBLN2 in molecular subtypes of human breast cancer. **a**–**j** *Fbln2* mRNA expression in different classification of human breast cancer [molecular subtypes, ER status, PR status, Her2 status, LN status, tumor grade, and tumor proliferation]. *P < 0.05, **P < 0.01, ***P < 0.001. G1: grade 1, G2: grade 2, G3: grade 3, ER + Her2− HP: ER + her2− High proliferation, ER + Her2− LP: ER + Her2− Low Proliferation, IDC: Invasive Ductal Carcinoma, ILC: Invasive Lobular Carcinoma, IMM: Invasive Mixed Mucinous, IMDLC: Invasive Mixed Ductal and Lobular Carcinoma, and IBC: Invasive Breast Carcinoma

We have previously reported that FBLN2 contributes to BM integrity in mouse mammary gland and human breast cancer [14]. Retrospective analysis of a previously examined cohort of 65 human breast cancer patients [14] revealed that FBLN2 generally had a higher expression in control regions (safety margins) compared to DCIS and IDC. Further, Her2 + and triple-negative patients had a higher expression of FBLN2 compared to LumA and LumB at lesions of invasive ductal carcinoma (IDC), ductal carcinoma in situ (DCIS), and control regions (Supplementary Fig. 7). To test these observations in a larger dataset, we retrieved human breast cancer mRNA profiles from the datasets METABRIC and TCGA, registered at the Bioportal [28–31], with stratification of patients according to their molecular and clinical subtypes. In METABRIC dataset, *Fbln2* was significantly upregulated in Claudin-low compared to LumA (*P* < 0.0001); however, it was upregulated in LumA when compared to Her2 + (*P* < 0.001) and LumB (*P* < 0.0001) (Fig. 5a).

In relation to hormonal and growth factor receptors, ER-patients showed a significant increase in *Fbln2* expression when compared to ER + patients (*P* < 0.0001) (Fig. 5b); however, no preferential expression was observed based on PR and Her-2 status in this dataset (Fig. 5c and d). According to tumor grade, *Fbln2* expression was significantly decreased in grade 3 compared to grade 1 (*P* < 0.01) and grade 2 (*P* < 0.05) (Fig. 5e). *Fbln2* expression did not significantly change upon subgrouping based on LN status (Fig. 5f). Patients with higher tumor proliferation ER + Her2- showed a significant downregulation of *Fbln2* compared to Lower proliferation group (*P* < 0.0001) (Fig. 5g). Based on pathological classification of patients, *Fbln2* was significantly downregulated in IDC patients when compared to invasive lobular carcinoma (ILC) (*P* < 0.0001). Invasive mixed mucinous breast cancer showed a significant downregulation of *Fbln2* compared to IDC (*P* < 0.001), ILC (*P* < 0.0001), mixed ductal carcinoma (*P* < 0.001), and invasive breast cancer (*P* < 0.05) (Fig. 5h).

These patterns of expression appeared to be conserved when analyzing the TCGA dataset (Molecular subtypes, ER status, PR status, and LN status); however, Her2− patients had a significantly lower expression of *Fbln2* compared to Her2 + patients (Supplementary Fig. 8). Our examination of METABRIC dataset showed that the expression patterns of *Krt14*, *Itgβ1*, and *Itgα3* were the same patterns as *Fbln2* expression in molecular subtypes ensuring the association between *fbln2* and basal markers (Supplementary Fig. 1). Interestingly, methylation status of the *Fbln2* promotor across molecular subtypes supports the different expression patterns of *Fbln2* (Supplementary Fig. 9), suggesting that expression of FBLN2 may be epigenetically regulated.

### FBLN2 expression in human breast cancer cell lines

**Fig. 6:**
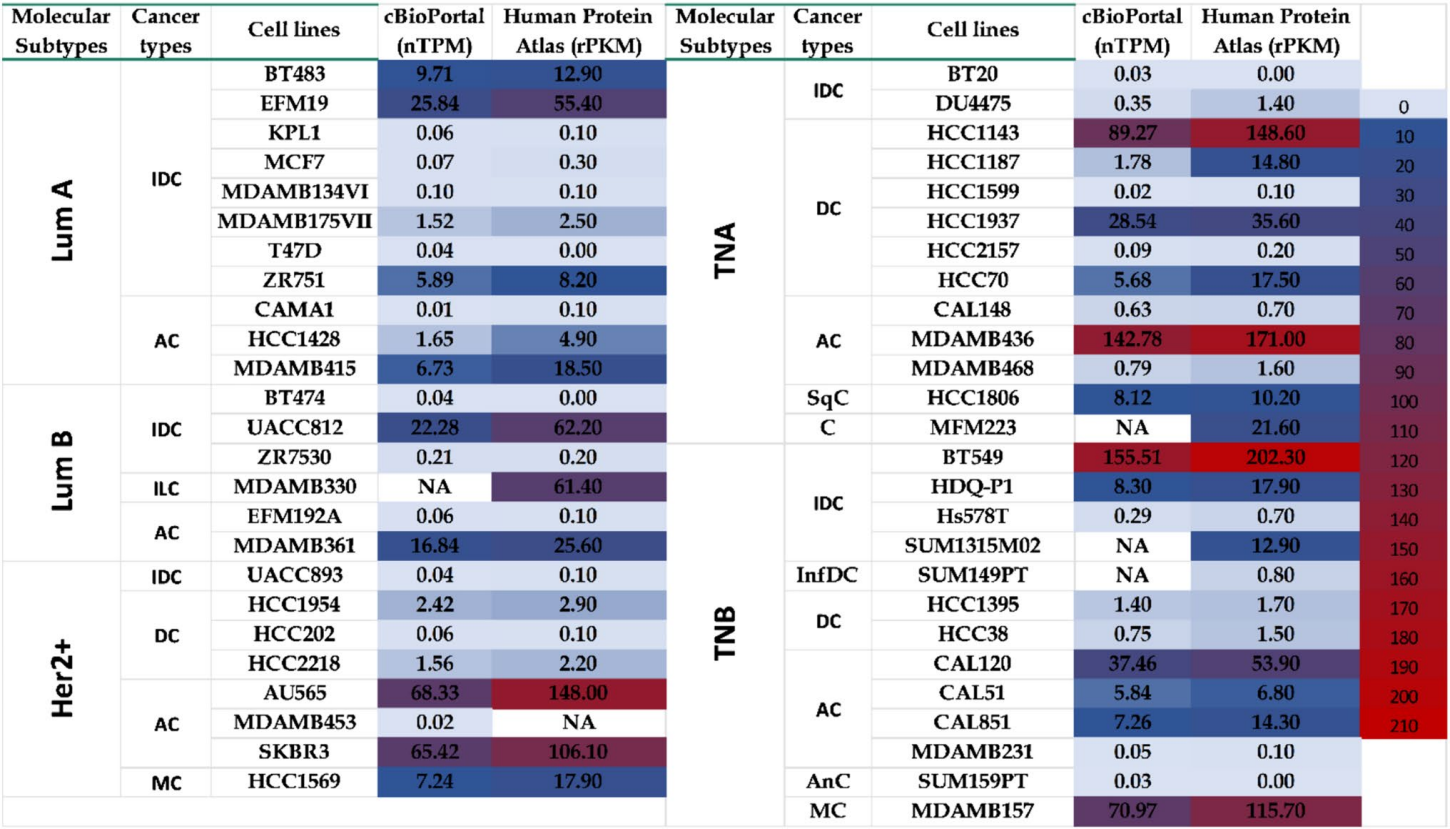
FBLN2 expression in human breast cancer cell lines. Heatmap of *Fbln2* mRNA expression levels in different human breast cancer cell lines categorized based on their molecular subtypes and pathological classification. nTPM: Normalized transcript per million, rPKM: Reads per kilobase per million mapped reads, IDC: Invasive ductal carcinoma, ILC: Incasive Lobular Carcinoma, AC: Adenocarcinoma, MC: Medullary carcinoma, Sqc: Squamous cell carcinoma, infDC: Inflammatory ductal carcinoma, and AnC: anaplastic carcinoma

To investigate FBLN2 expression in the different molecular subtypes of human breast cancer, meta-analyses of publicly available datasets of breast cancer cell lines from Bioportal [28–31] and the Human Protein Atlas [35] revealed that FBLN2 had a lower expression in cell lines that represent LumA and LumB subtypes and had a higher expression in cell lines that represent Her2 + and triple-negative subtypes (Fig. 6).

### Higher FBLN2 expression is associated with better survival in less advanced bbreast cancer

**Fig. 7:**
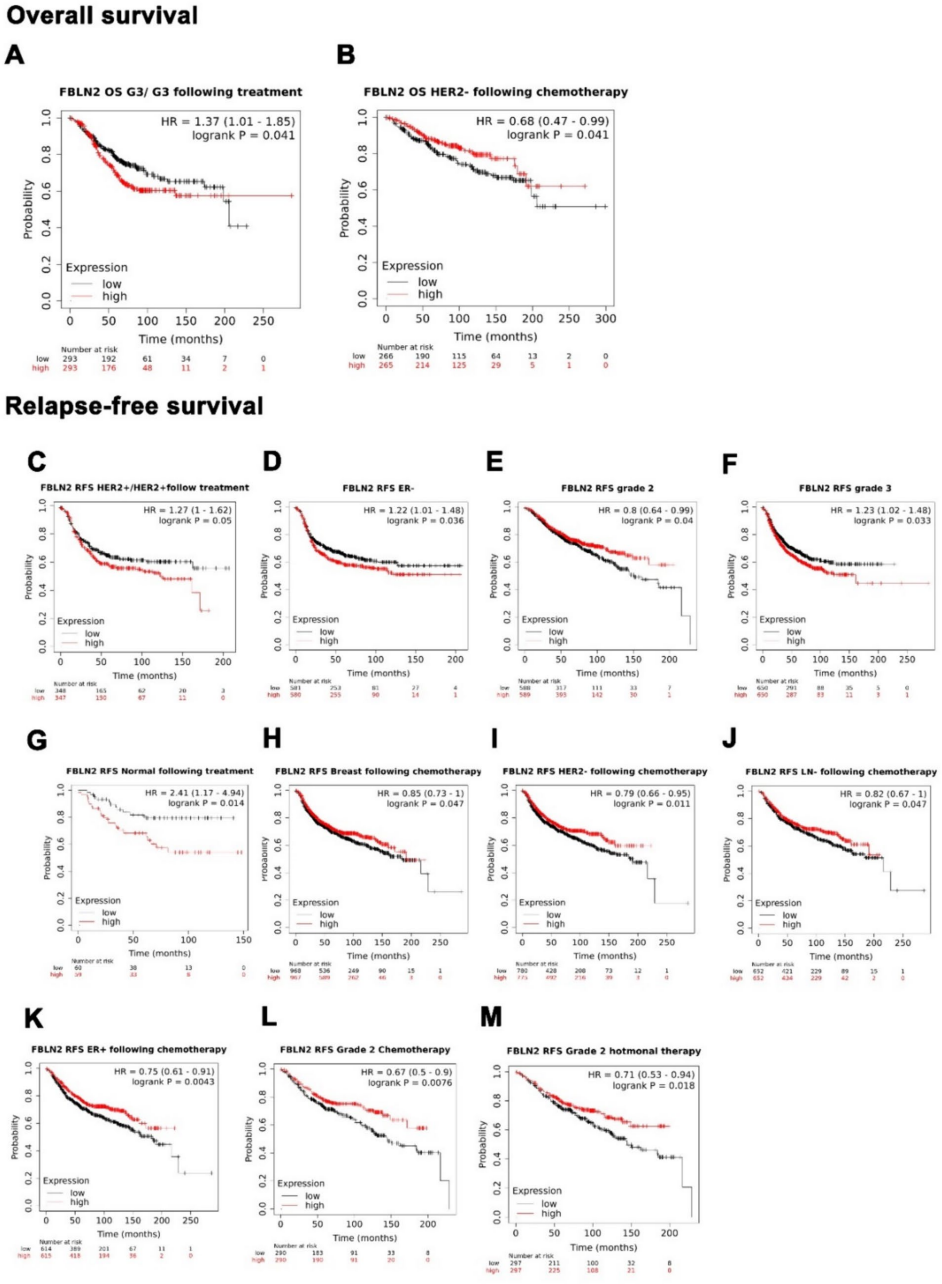
Higher *Fbln2* mRNA expression is associated with better survival in less advanced human breast cancer. KM plots for overall survival (**a** and **b**), and Relapse-free survival (**c–m**) based on *Fbln2* expression levels, showing the stratification of breast cancer patients based on grade, Her2 status, LN status, and therapy regimen. High mRNA expression in red and low expression in black. The numbers below each plot are the numbers of individuals at risk in each group

To evaluate the prognostic potential of FBLN2 expression in breast cancer patients, we assessed the relevance of *Fbln2* mRNA expression to patients’ survival status using data from the KM plotter and METABRIC. With multiple analyses in KM plotter dataset, higher *Fbln2* expression was significantly associated with better OS in patients with Her2− status (*P* = 0.041, *n* = 531) following chemotherapy. RFS improved with higher *Fbln2* expression in grade 2 patients (*P* = 0.04, *n* = 1177), unstratified breast cancer patients following chemotherapy (*P* = 0.047, *n* = 1935), Her2− patients following chemotherapy (*P* = 0.011, *n* = 1555), LN-patients following chemotherapy (*P* = 0.047, *n* = 1304), ER + patients following chemotherapy (*P* = 0.0043, *n* = 1229), grade 2 patients following chemotherapy (*P* = 0.0076, *n* = 580), and grade 2 patients following hormonal therapy (*P* = 0.018, *n* = 594). In contrast, lower *Fbln2* expression was associated with better OS in grade 3 patients (*P* = 0.041, *n* = 586). RFS improved with lower expression of *Fbln2* in Her2 + patients (*P* = 0.049, *n* = 695), ER-patients (*P* = 0.036, *n* = 1161), grade 3 patients (*P* = 0.033, *n* = 1300), normal following treatment (*P* = 0.014, *n* = 119), and Her2 + patients following treatment (*P* = 0.05, *n* = 695) (Fig. 7).

In TCGA dataset, lower *Fbln2* expression was associated with better OS in LumB patients (*P* < 0.05), and higher *Fbln2* expression was associated with better OS in Her2 + patients (*P* < 0.01) (Supplementary Fig. 10).

## Discussion

We have recently demonstrated that FBLN2 is preferentially expressed in TEB and not in the ductal epithelium during puberty, and in the myoepithelial cells of ducts during early pregnancy, at times of epithelial morphogenesis [13, 14]. We have further reported that FBLN2 contributes to BM integrity during mouse mammary epithelial morphogenesis and in human breast cancer [14]. Herein, our results confirmed the association of FBLN2 to myoepithelial phenotype in pubertal mouse mammary gland and mouse mammary epithelial cells. We further translated these results to human datasets by exploring *fbln2* mRNA expression in human cell lines which confirmed the association of fbln2 with basal molecular subtypes. Further we presented the correlation of *fbln2* with myoepithelial and basal cell markers in different molecular subtypes with a preferential expression in basal and claudin-low molecular subtypes.

Cell–ECM interaction is influential in regulating structural and functional features of mammary epithelial cells and stromal activity during normal development and cancer progression [12, 32, 48–50]. Therefore, it is crucial to disseminate the ECM composition of mammary and breast cancer microenvironment. We have reported that *Fbln2* KD results in reduction of ITGβ1 in mammary epithelial cells, associated with a disruption in COL IV sheath surrounding mammary cells in vitro [14]. The current study further confirms the association between FBLN2 and ITGβ1 in puberal mouse mammary gland, particularly with preferential expression in cap cells of TEBs. The association between FBLN2 and ITGβ1 has also been reported during BM homeostasis of mouse skin [51]. ITGβ1 is crucial for maintaining the population of functional stem cells, mammary morphogenesis, and lineage segregation of mammary cells [52], which suggests relevance of FBLN2 to basal/mesenchymal and stemness phenotype in mammary gland. A notion that was further confirmed through retrieving *Fbln2* expression data from Giraddi’s dataset [47] (Supplementary data 6). ITG-α3β1 was suggested to control tumor invasion and growth in MMTV-cNeu mouse model [53]. The co-localization of FBLN2 with integrins α3 and β1 suggests its importance in cap cells function during normal mammary morphogenesis, particularly with cell–ECM interaction-based invasion of the surrounding fat pad. Our data further report an upregulation of FBLN2 upon stimulation with TGFβ3 in a mammary epithelial cell line, EpH4 cells, and in MEFs. TGFβ signaling is crucial for mammary gland development via balancing proliferation and morphogenesis [54], via stimulating apoptosis, ECM remodeling, and suppression of proliferation in mouse mammary gland [55, 56]. It has previously been suggested that FBLN2 is required for the activation of TGFβ signaling in cardiac tissue [10]. Here, we report that this activation can be reciprocal in mouse mammary cells, which further supports that FBLN2 may potentially contribute to controlled mammary invasion during epithelial morphogenesis.

In human breast cancer, we show a positive correlation of *Fbln2* mRNA with basal markers (mesenchymal and myoepithelial) and a negative correlation with luminal markers. We, therefore, hypothesized that this preferential correlation can affect FBLN2 expression in molecular subtypes, particularly with the heterogeneous composition of tumor microenvironment [57, 58]. Of note, *Fbln2* mRNA had a strong positive correlation with *Fbln1* and *Fbln5*, which agrees with previous reports on their cross-compensation and sharing binding partners [13, 59].

In a previous study, FBLN2 has been poorly expressed in seven human breast cancer cell lines where KRT19 (luminal marker) was highly expressed [60]. In our analyses of multiple datasets, *Fbln2* mRNA had a higher expression in normal-like and lumA breast cancers compared to LumB, Her2 + , and Basal-like. *Fbln2* also showed a higher expression in claudin-low compared to LumA and other subtypes. This expression pattern could partially be attributed to the different expression levels of basal markers in each subtype and indeed the level of breast cancer development. For instance, Claudin-low subtypes represent basal-like phenotype, with an increased EMT, immune response, and cancer stem cell (CSC) signatures [61]. Claudin-low tumors express variable keratin expression (keratins 5, 14, and 17), with low expression of Her2 and luminal markers such ER, PR, GATA3, Krt18 and Krt19, as well as the luminal gene cluster [62]. LumA tumors are ER + , PR + , and Her2 − , with a low proliferation index [25, 39]. KRT18 and KRT19 were positively correlated with LumA, while KRT14, KRT5, and KRT6 were negatively correlated [40]. In different molecular subtypes, perivascular FBLN2 has shown a preferential expression in Luminal breast cancer subtypes compared to basal and Her2 + subtypes [63]. Further, the high expression of *Fbln2* in LumA may be originated from the maintenance of BM and/or ECM surrounding the tumor cells as previously described [14].

LumB has a higher proliferation index compared to LumA [39], with a reported high expression of KRT18 and KRT19 [40]. This may explain the lower expression of *Fbln2* in LumB tumors compared to LumA, as *Fbln2* appears to be associated with low proliferative tumors, according to our analysis. In contrast, Basal and Her2 showed a higher expression compared to LumB, but not Lum A, which suggests an increased expression of *Fbln2* in tumor cells, particularly with EMT, myoepithelial turnover, and tumor invasion [64–66]. These observations in human breast cancer subtypes were further supported by protein and transcriptome data from the Human Protein Atlas and Bioportal repository, respectively. Further investigations are required to disseminate the functional expression of FBLN2 in each subtype, and the epithelial versus stromal expression of FBLN2 in tumor microenvironment. Our analyses of publicly available datasets revealed an inverse correlation between *Krt18* and *Fbln2* expression across molecular subtypes. Analysis of FBLN2 methylation status in METABRIC dataset showed the least methylation in basal-like breast cancer (Supplementary Fig. 9), which agrees with a recent report showing an increased metastasis and invasion with methylation of *Fbln2* in breast cancer [67, 68]. It is crucial, however, to disseminate the role of FBLN2 in tumor cells and BM/ECM at early versus advanced stages of breast cancer.

Survival analysis of *Fbln2* showed different prognosis of breast cancer at different stages of tumor development, where higher *Fbln2* was associated with a better prognosis in early stages; however, low *Fbln2* was associated with a better prognosis in advanced breast cancer. This agrees with the notion that FBLN2 can potentially have a dual role as a cancer promotor or inhibitor based on tumor status [69, 70]. We have previously reported that FBLN2 expression in DCIS (relatively early stage of cancer progression [14]) can play a protective role against BM disruption, and therefore, high expression is advantageous [13, 14]. This indeed is shown to be reversed in later stages, particularly during metastasis, that may confer a growth advantage for metastatic cells to invade and establish a niche for tumor growth. A similar mechanism has been proposed in lung adenocarcinoma, where FBLN2 can facilitate the development of the new ECM surrounding tumor cells, perhaps originating from tumor-associated fibroblasts [71].

The limitation of this study is the utilization of *Fbln2* mRNA data, and not protein data, from publicly available datasets that represent whole tissue transcriptome with no dissection of tumor versus microenvironment expression.

In conclusion, FBLN2 is associated with basal cell markers and exhibits different expression patterns in molecular subtypes, with the highest mRNA expression in Claudin-low tumors. This may offer a promising molecular tool for patients’ prognosis for more personalized therapeutic approaches.

## Supplementary Information

Below is the link to the electronic supplementary material.

Supplementary file1 (DOCX 4727 KB)

Supplementary file2 (XLSX 25 KB)

## Data Availability

The datasets used and/or analyzed during the current study available from the corresponding author on reasonable request.

## References

[1] Ferlay J et al (2019) Estimating the global cancer incidence and mortality in 2018: GLOBOCAN sources and methods. Int J Cancer 144:1941–1953 30350310 10.1002/ijc.31937

[2] Momenimovahed Z, Salehiniya H (2019) Epidemiological characteristics of and risk factors for breast cancer in the world. Breast Cancer Targets Ther 11:151–164 10.2147/BCTT.S176070 PMC6462164 31040712

[3] Lüönd F, Tiede S, Christofori G (2021) Breast cancer as an example of tumour heterogeneity and tumour cell plasticity during malignant progression. Br J Cancer 125:164–175 33824479 10.1038/s41416-021-01328-7 PMC8292450

[4] Bissell MJ, Kenny PA, Radisky DC (2005) Microenvironmental regulators of tissue structure and function also regulate tumor induction and progression: the role of extracellular matrix and its degrading enzymes. Cold Spring Harb Symp Quant Biol 70:343–356 16869771 10.1101/sqb.2005.70.013 PMC3004779

[5] Kalluri R, Zeisberg M (2006) Fibroblasts in cancer. Nat Rev Cancer 6:392–401 16572188 10.1038/nrc1877

[6] Mouw JK, Ou G, Weaver VM (2014) Extracellular matrix assembly: a multiscale deconstruction. Nat Rev Mol Cell Biol 15:771–785 25370693 10.1038/nrm3902 PMC4682873

[7] Cirri P, Chiarugi P (2012) Cancer-associated-fibroblasts and tumour cells: a diabolic liaison driving cancer progression. Cancer Metastasis Rev 31:195–208 22101652 10.1007/s10555-011-9340-x

[8] Cox TR, Erler JT (2011) Remodeling and homeostasis of the extracellular matrix: implications for fibrotic diseases and cancer. Dis Model Mech 4:165–178 21324931 10.1242/dmm.004077 PMC3046088

[9] Ibrahim AM et al (2020) Diverse macrophage populations contribute to the inflammatory microenvironment in premalignant lesions during localized invasion. Front Oncol 10:569985 33072601 10.3389/fonc.2020.569985 PMC7541939

[10] Ibrahim AM et al (2020) An investigation of fibulin-2 in hypertrophic cardiomyopathy. Int J Mol Sci 21:1–14 10.3390/ijms21197176 PMC7583916 33003281

[11] Ibrahim AM et al (2023) Structural and functional characterization of the aorta in hpertrophic obstructive cardiomyopathy. medRxiv 2023.05.23.23290086. 10.1161/CIRCHEARTFAILURE.124.012384 PMC11832182 39846175

[12] Ibrahim AM, Bilsland A, Rickelt S, Morris JS, Stein T (2021) A matrisome RNA signature from early-pregnancy mouse mammary fibroblasts predicts distant metastasis-free breast cancer survival in humans. Breast Cancer Res 23:90 34565423 10.1186/s13058-021-01470-3 PMC8474794

[13] Olijnyk D et al (2014) Fibulin-2 is involved in early extracellular matrix development of the outgrowing mouse mammary epithelium. Cell Mol Life Sci 71:3811–3828 24522256 10.1007/s00018-014-1577-4 PMC11113845

[14] Ibrahim AM et al (2018) Fibulin-2 is required for basement membrane integrity of mammary epithelium. Sci Rep 8:1–14 30237579 10.1038/s41598-018-32507-x PMC6148073

[15] Grässel S, Sicot FX, Gotta S, Chu ML (1999) Mouse fibulin-2 gene. Complete exon-intron organization and promoter characterization. Eur J Biochem 263:471–477. 10.1046/j.1432-1327.1999.00523.x 10406956

[16] Utani A, Nomizu M, Yamada Y (1997) Fibulin-2 binds to the short arms of laminin-5 and laminin-1 via conserved amino acid sequences. J Biol Chem 272:2814–2820 9006922 10.1074/jbc.272.5.2814

[17] Talts JF, Andac Z, Göhring W, Brancaccio A, Timpl R (1999) Binding of the G domains of laminin alpha1 and alpha2 chains and perlecan to heparin, sulfatides, alpha-dystroglycan and several extracellular matrix proteins. EMBO J 18:863–870 10022829 10.1093/emboj/18.4.863 PMC1171179

[18] Reinhardt DP et al (1996) Fibrillin-1 and fibulin-2 interact and are colocalized in some tissues. J Biol Chem 271:19489–19496 8702639 10.1074/jbc.271.32.19489

[19] Ng K-M et al (2006) Evidence that fibulin family members contribute to the steroid-dependent extravascular sequestration of sex hormone-binding globulin. J Biol Chem 281:15853–15861 16601122 10.1074/jbc.M512370200

[20] Sasaki T, Göhring W, Pan TC, Chu ML, Timpl R (1995) Binding of mouse and human fibulin-2 to extracellular matrix ligands. J Mol Biol 254:892–899 7500359 10.1006/jmbi.1995.0664

[21] Hopf M, Göhring W, Kohfeldt E, Yamada Y, Timpl R (1999) Recombinant domain IV of perlecan binds to nidogens, laminin-nidogen complex, fibronectin, fibulin-2 and heparin. Eur J Biochem 259:917–925 10092882 10.1046/j.1432-1327.1999.00127.x

[22] Brown JC, Sasaki T, Göhring W, Yamada Y, Timpl R (1997) The C-terminal domain V of perlecan promotes beta1 integrin-mediated cell adhesion, binds heparin, nidogen and fibulin-2 and can be modified by glycosaminoglycans. Eur J Biochem 250:39–46 9431988 10.1111/j.1432-1033.1997.t01-1-00039.x

[23] Pérez-Rico C et al (2011) Tropoelastin and fibulin overexpression in the subepithelial connective tissue of human pterygium. Am J Ophthalmol 151:44–52 21094933 10.1016/j.ajo.2010.07.012

[24] Zhang HY, Timpl R, Sasaki T, Chu ML, Ekblom P (1996) Fibulin-1 and fibulin-2 expression during organogenesis in the developing mouse embryo. Dev Dyn Off Publ Am Assoc Anat 205:348–364. 10.1002/(SICI)1097-0177(199603)205:3<348::AID-AJA13>3.0.CO;2-0 8850569

[25] Yip CH et al (2016) Roles of Ki67 in breast cancer—important for management? Asian Pacific J Cancer Prev 17:1077–1082 10.7314/apjcp.2016.17.3.1077 27039727

[26] Goldhirsch A et al (2011) Strategies for subtypes-dealing with the diversity of breast cancer: highlights of the St Gallen international expert consensus on the primary therapy of early breast cancer 2011. Ann Oncol 22:1736–1747 21709140 10.1093/annonc/mdr304 PMC3144634

[27] Wang J et al (2016) Value of breast cancer molecular subtypes and ki67 expression for the prediction of efficacy and prognosis of neoadjuvant chemotherapy in a Chinese population. Med (United States) 95:e3518. 10.1097/MD.0000000000003518 PMC4863770 27149453

[28] Rueda OM et al (2019) Dynamics of breast-cancer relapse reveal late-recurring ER-positive genomic subgroups. Nature 567:399–404 30867590 10.1038/s41586-019-1007-8 PMC6647838

[29] Pereira B et al (2016) The somatic mutation profiles of 2,433 breast cancers refines their genomic and transcriptomic landscapes. Nat Commun 7:11479 27161491 10.1038/ncomms11479 PMC4866047

[30] Curtis C et al (2012) The genomic and transcriptomic architecture of 2,000 breast tumours reveals novel subgroups. Nature 486:346–352 22522925 10.1038/nature10983 PMC3440846

[31] Cancer Genome Atlas Network (2012) Comprehensive molecular portraits of human breast tumours. Nature 490:61–70. 10.1038/nature11412 10.1038/nature11412 PMC3465532 23000897

[32] Ibrahim AM et al (2020) Gas6 expression is reduced in advanced breast cancers. NPJ Precis Oncol 4:9. 10.1038/s41698-020-0116-z PMC7181799 32352034

[33] Ghandi M et al (2019) Next-generation characterization of the cancer cell line encyclopedia. Nature 569:503–508 31068700 10.1038/s41586-019-1186-3 PMC6697103

[34] Nusinow DP et al (2020) Quantitative proteomics of the cancer cell line encyclopedia. Cell 180:387-402.e16 31978347 10.1016/j.cell.2019.12.023 PMC7339254

[35] Uhlén M et al (2005) A human protein atlas for normal and cancer tissues based on antibody proteomics. Mol Cell Proteomics 4:1920–1932 16127175 10.1074/mcp.M500279-MCP200

[36] Győrffy B (2021) Survival analysis across the entire transcriptome identifies biomarkers with the highest prognostic power in breast cancer. Comput Struct Biotechnol J 19:4101–4109 34527184 10.1016/j.csbj.2021.07.014 PMC8339292

[37] Nguyen QH et al (2018) Profiling human breast epithelial cells using single cell RNA sequencing identifies cell diversity. Nat Commun 9:2028 29795293 10.1038/s41467-018-04334-1 PMC5966421

[38] Franzén O, Gan L-M, Björkegren JLM (2019) PanglaoDB: a web server for exploration of mouse and human single-cell RNA sequencing data. Database, baz046. 10.1093/database/baz046 PMC6450036 30951143

[39] Vuong D, Simpson PT, Green B, Cummings MC, Lakhani SR (2014) Molecular classification of breast cancer. Virchows Arch 465:1–14 24878755 10.1007/s00428-014-1593-7

[40] Shao MM et al (2012) Keratin expression in breast cancers. Virchows Arch 461:313–322 22851038 10.1007/s00428-012-1289-9

[41] Paine IS, Lewis MT (2017) The terminal end bud: the little engine that could. J Mammary Gland Biol Neoplasia 22:93–108 28168376 10.1007/s10911-017-9372-0 PMC5488158

[42] Walker LC et al (2007) Cytokeratin KRT8/18 expression differentiates distinct subtypes of grade 3 invasive ductal carcinoma of the breast. Cancer Genet Cytogenet 178:94–103 17954264 10.1016/j.cancergencyto.2007.06.002

[43] Moll R, Franke WW, Schiller DL, Geiger B, Krepler R (1982) The catalog of human cytokeratins: patterns of expression in normal epithelia, tumors and cultured cells. Cell 31:11–24 6186379 10.1016/0092-8674(82)90400-7

[44] Abd El-Rehim DM et al (2004) Expression of luminal and basal cytokeratins in human breast carcinoma. J Pathol 203:661–671. 10.1002/path.1559 15141381

[45] Huang YH et al (2021) Expression pattern and prognostic impact of glycoprotein non-metastatic B (GPNMB) in triple-negative breast cancer. Sci Rep 11:1–12 34108545 10.1038/s41598-021-91588-3 PMC8190094

[46] Herreros-Pomares A (2022) Identification, culture and targeting of cancer stem cells. Life (Basel, Switzerland), 12. 10.3390/life12020184 PMC8879966 35207472

[47] Giraddi RR et al (2018) Single-cell transcriptomes distinguish stem cell state changes and lineage specification programs in early mammary gland development. Cell Rep 24:1653-1666.e7 30089273 10.1016/j.celrep.2018.07.025 PMC6301014

[48] Muschler J, Streuli CH (2010) Cell-matrix interactions in mammary gland development and breast cancer. Cold Spring Harb Perspect Biol 2:1–17 10.1101/cshperspect.a003202 PMC2944360 20702598

[49] Moumen M et al (2011) The mammary myoepithelial cell. Int J Dev Biol 55:763–771 21948739 10.1387/ijdb.113385mm

[50] Wang Y et al (2020) Tissue-resident macrophages promote extracellular matrix homeostasis in the mammary gland stroma of nulliparous mice. Elife, 9. 10.7554/eLife.57438 PMC7297528 32479261

[51] Longmate WM et al (2014) Reduced fibulin-2 contributes to loss of basement membrane integrity and skin blistering in mice lacking integrin α3β1 in the epidermis. J Invest Dermatol 134:1609–1617 24390135 10.1038/jid.2014.10 PMC4020984

[52] Taddei I et al (2008) Beta1 integrin deletion from the basal compartment of the mammary epithelium affects stem cells. Nat Cell Biol 10:716–722 18469806 10.1038/ncb1734 PMC2659707

[53] Ramovs V et al (2019) Absence of integrin α3β1 promotes the progression of HER2-driven breast cancer in vivo. Breast Cancer Res 21:1–17 31101121 10.1186/s13058-019-1146-8 PMC6525362

[54] Moses H, Barcellos-Hoff MH (2011) TGF-β Biology in mammary development and breast cancer. Cold Spring Harb Perspect Biol 3:1–14 10.1101/cshperspect.a003277 PMC3003461 20810549

[55] Ewan KBR et al (2005) Proliferation of estrogen receptor-alpha-positive mammary epithelial cells is restrained by transforming growth factor-beta1 in adult mice. Am J Pathol 167:409–417 16049327 10.1016/s0002-9440(10)62985-9 PMC1603552

[56] Ewan KB et al (2002) Latent transforming growth factor-beta activation in mammary gland: regulation by ovarian hormones affects ductal and alveolar proliferation. Am J Pathol 160:2081–2093 12057913 10.1016/s0002-9440(10)61158-3 PMC1850834

[57] Kashyap D et al (2023) Heterogeneity of the tumor microenvironment across molecular subtypes of breast cancer. Appl Immunohistochem Mol Morphol AIMM 31:533–543 37358863 10.1097/PAI.0000000000001139

[58] Li J, Wu J, Han J (2023) Analysis of tumor microenvironment heterogeneity among breast cancer subtypes to identify subtype-specific signatures. Genes (Basel), 14. 10.3390/genes14010044 PMC9858482 36672784

[59] Timpl R, Sasaki T, Kostka G, Chu M-L (2003) Fibulins: a versatile family of extracellular matrix proteins. Nat Rev Mol Cell Biol 4:479–489 12778127 10.1038/nrm1130

[60] Yi C-H, Smith DJ, West WW, Hollingsworth MA (2007) Loss of fibulin-2 expression is associated with breast cancer progression. Am J Pathol 170:1535–1545 17456760 10.2353/ajpath.2007.060478 PMC1854949

[61] Turner KM, Yeo SK, Holm TM, Shaughnessy E, Guan J-L (2021) Heterogeneity within molecular subtypes of breast cancer. Am J Physiol Cell Physiol 321:C343–C354 34191627 10.1152/ajpcell.00109.2021 PMC8424677

[62] Prat A et al (2010) Phenotypic and molecular characterization of the claudin-low intrinsic subtype of breast cancer. Breast Cancer Res 12:R68 20813035 10.1186/bcr2635 PMC3096954

[63] Klingen TA, Chen Y, Aas H, Wik E, Akslen LA (2021) Fibulin-2 expression associates with vascular invasion and patient survival in breast cancer. PLoS ONE 16:1–14 10.1371/journal.pone.0249767 PMC8034712 33836007

[64] Law EWL et al (2012) Anti-angiogenic and tumor-suppressive roles of candidate tumor-suppressor gene, Fibulin-2, in nasopharyngeal carcinoma. Oncogene 31:728–738 21743496 10.1038/onc.2011.272

[65] Baird BN et al (2013) Fibulin-2 is a driver of malignant progression in lung adenocarcinoma. PLoS ONE 8:e67054 23785517 10.1371/journal.pone.0067054 PMC3677922

[66] Sofela AA et al (2021) Fibulin-2: a novel biomarker for differentiating grade II from grade I meningiomas. Int J Mol Sci, 22. 10.3390/ijms22020560 PMC7827565 33429944

[67] Ma Y et al (2021) Fibulin 2 is hypermethylated and suppresses tumor cell proliferation through inhibition of cell adhesion and extracellular matrix genes in non-small cell lung cancer. Int J Mol Sci, 22. 10.3390/ijms222111834 PMC8584407 34769264

[68] Hill VK et al (2010) Identification of 5 novel genes methylated in breast and other epithelial cancers. Mol Cancer 9:51 20205715 10.1186/1476-4598-9-51 PMC2841122

[69] Obaya AJ, Rua S, Moncada-Pazos A, Cal S (2012) The dual role of fibulins in tumorigenesis. Cancer Lett 325:132–138 22781395 10.1016/j.canlet.2012.06.019

[70] Zhang H, Hui D, Fu X (2020) Roles of fibulin-2 in carcinogenesis. Med Sci Monit 26:1–9 10.12659/MSM.918099 PMC6977632 31915327

[71] Baird BN et al (2013) Fibulin-2 is a driver of malignant progression in lung adenocarcinoma. PLoS ONE 8:1–10 10.1371/journal.pone.0067054 PMC3677922 23785517

