# Correction: FBLN2 is associated with basal cell markers Krt14 and ITGB1 in mouse mammary epithelial cells and has a preferential expression in molecular subtypes of human breast cancer

**DOI:** 10.1007/s10549-024-07472-x

**Published:** 2024-08-29

**Authors:** Amr Ahmed WalyEldeen, Salwa Sabet, Shady E. Anis, Torsten Stein, Ayman M. Ibrahim

**Affiliations:** 1 Department of Zoology, Faculty of Science, Cairo University, Giza, 12613 Egypt; 2 Department of Pathology, Faculty of Medicine, Cairo University, Cairo, 11562 Egypt; 3 Institute of Veterinary Biochemistry, Freie Universität Berlin, 14163 Berlin, Germany; 4 Institute of Cancer Sciences, College of MVLS, University of Glasgow, Glasgow, G12 8QQ UK; 5 Aswan Heart Centre, Magdi Yacoub Heart Foundation, Aswan, Egypt

**Correction to: Breast Cancer Research and Treatment** 10.1007/s10549-024-07447-y

In this article the affiliation “Institute of Veterinary, Freie Universität Berlin, 14163 Biochemistry Berlin, Germany” is published with a typo. It should have been “Institute of Veterinary Biochemistry, Freie Universität Berlin, 14163, Berlin, Germany”.

The original article has been corrected.

