# Supplementary material for: FBLN2 is associated with basal cell markers Krt14 and ITGB1 in mouse mammary epithelial cells and has a preferential expression in molecular subtypes of human breast cancer
Source: Breast Cancer Res Treat. 2024 Aug 7;208(3):673–86. doi: 10.1007/s10549-024-07447-y (PMC11522194; doi:10.1007/s10549-024-07447-y)
Supplement: Supplementary file 1 — Supplementary file1 (DOCX 4727 KB) [file 10549_2024_7447_MOESM1_ESM.docx]

# Supplementary Figures

**Supplementary Figure 1**


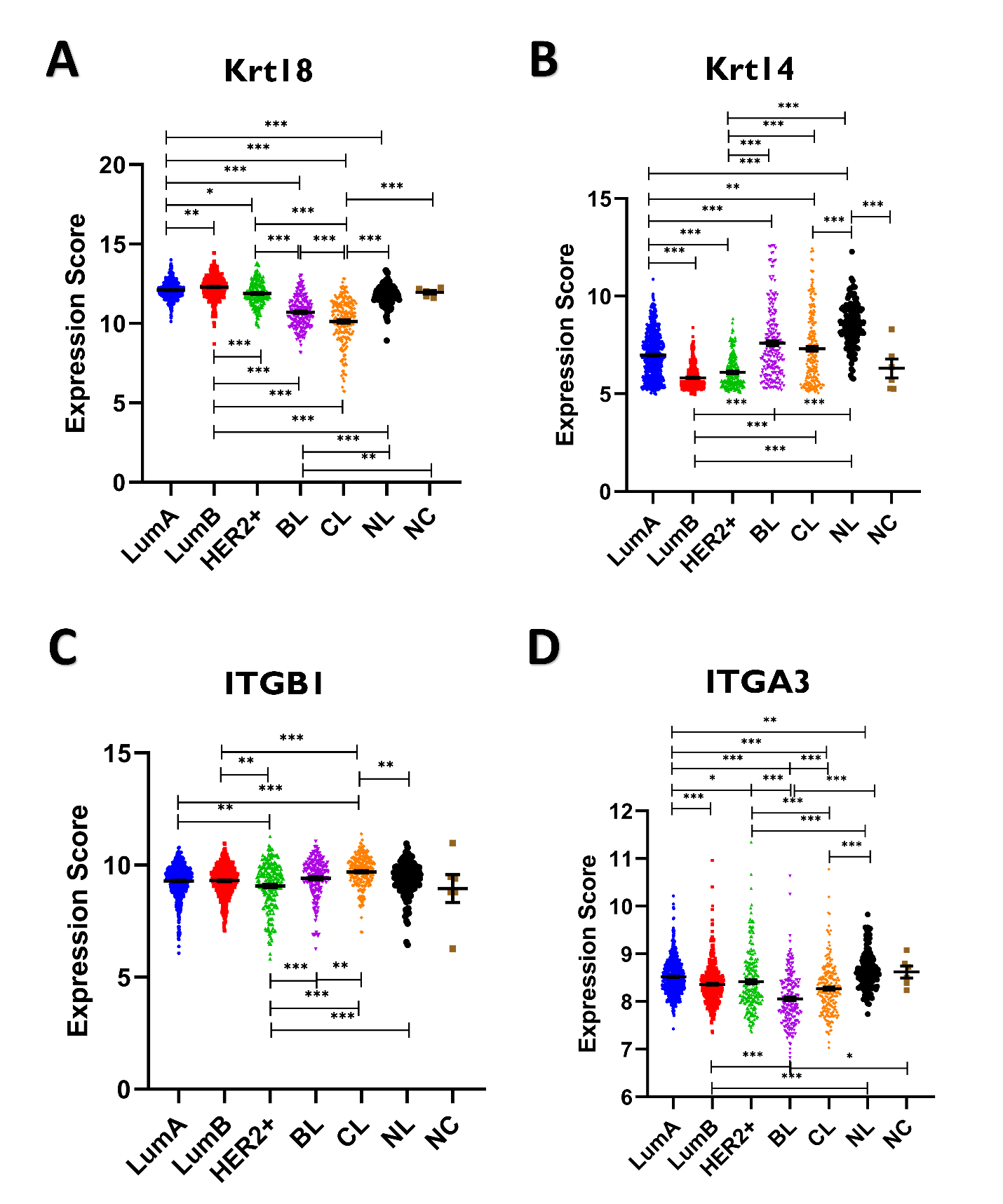


**Supplementary Figure 1.** Expression of Krt18, Krt14, Itgb1, and Itga3 across molecular subtypes of human breast cancer. LumA (n=700), LumB (n=475), Her2+ (n=224), Basal-Like (BL) (n=209), Claudin-Low (CL) (n=218), Normal-Like (NL) (n=148), and Non-Cancerous (NC) (n=6). *P < 0.05, **P < 0.01 AND *** P < 0.001.

**Supplementary Figure 2**


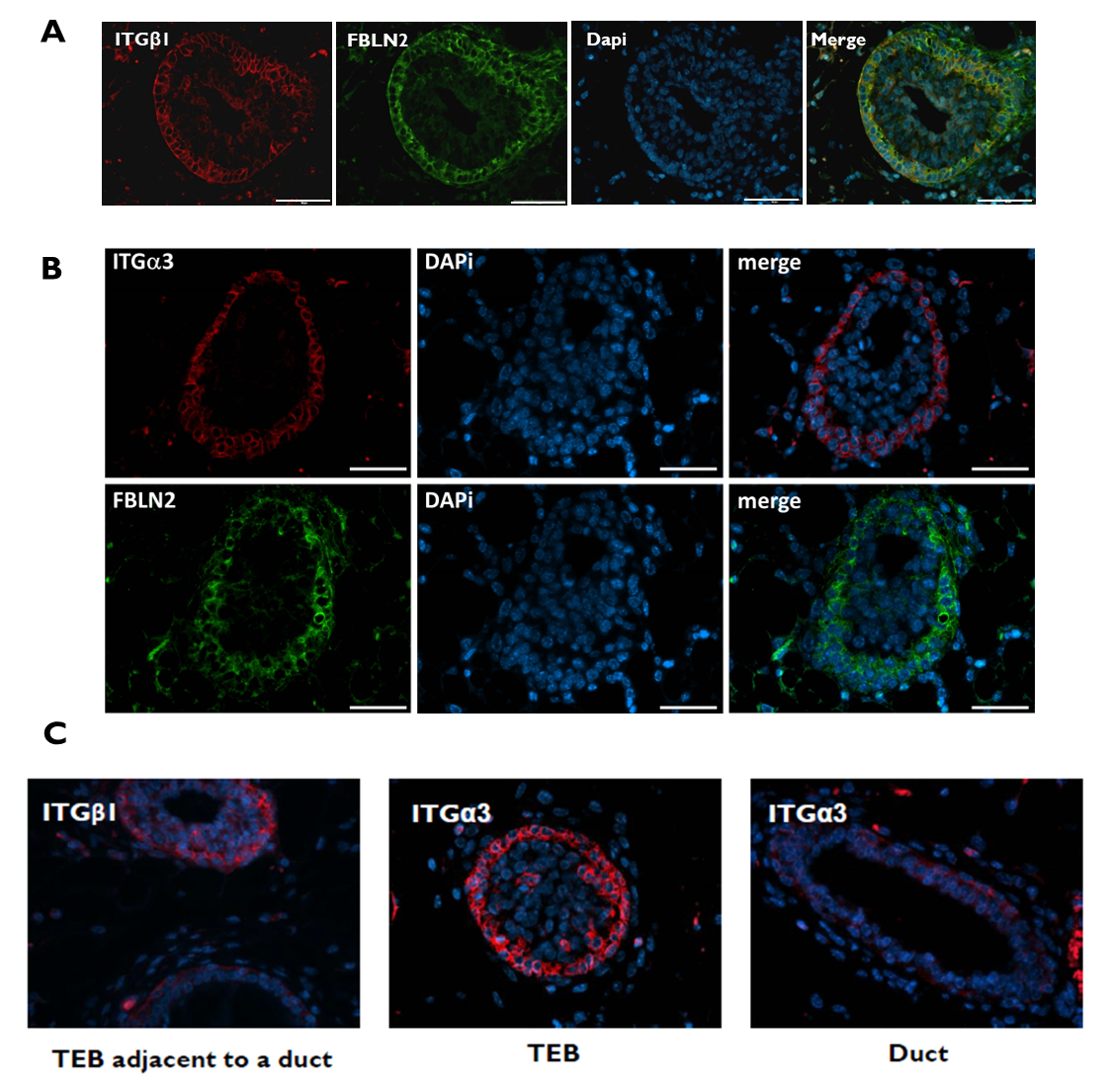


**Supplementary Figure 2. FBLN2 is co-localized with ITGβ1 and ITGα3 in the terminal end buds of pubertal mouse mammary gland in mice.** Immunofluorescence of pubertal mouse mammary gland showing the localization of ITGβ1 and ITGα3 at the TEBs and not in the ducts (n=3). Scale Bars represent 50μm.

**Supplementary Figure 3**

**
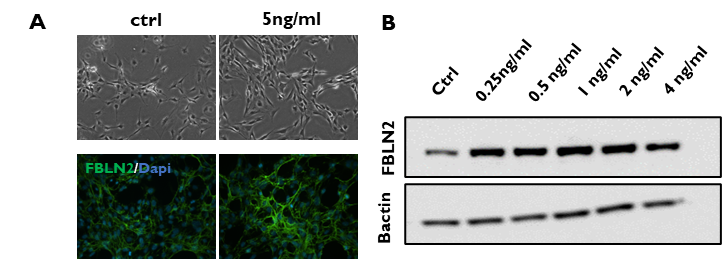
**

**Supplementary Figure 3: A:** Phase contrast and immunofluorescence imaging of ctrl MEFs vs TGFβ3-treated MEFs (n=1). **B:** Immunoblotting showing the upregulation of FBLN2 in MEFs upon TGFβ3 treatment in a dose-independent manner (n=1).

**Supplementary Figure 4**


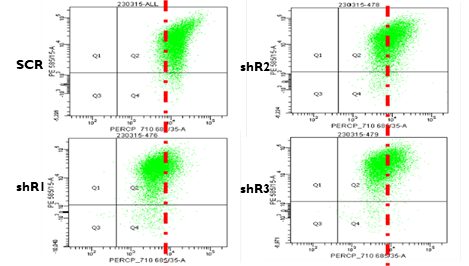


**Supplementary Figure 4:** Flow cytometry analysis showing the shift towards more luminal phenotype (CD24+) with FBLN2 downregulation. (n=1)

**
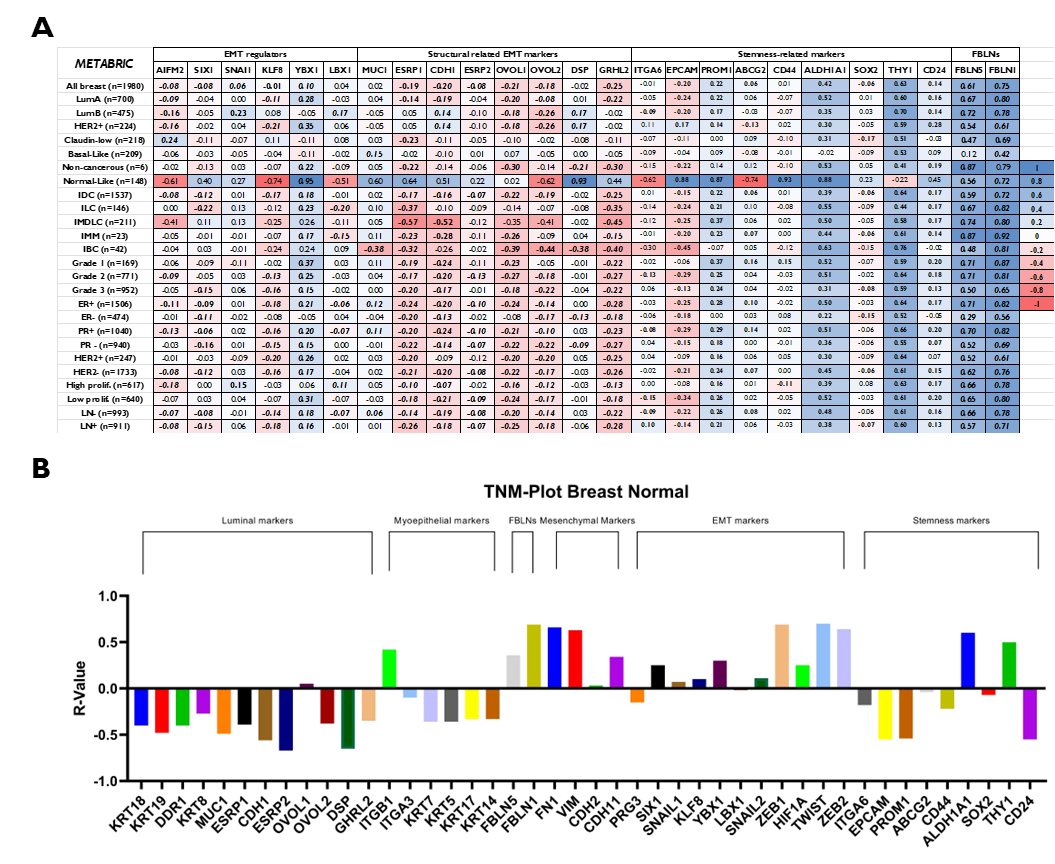
Supplementary Figure 5**

**Supplementary Figure 5: A:** Correlation matrix of data retrieved from online datasets (METABRIC) for Fbln2 against markers of EMT regulation, stemness markers and other Fblns. **B:** Correlation matrix generated by TNMplot.com for *Fbln2* with luminal, myoepithelial cell markers, EMT, mesenchymal, stemness and *Fblns1* and *5* in breast tumors.

**Supplementary Figure 6**


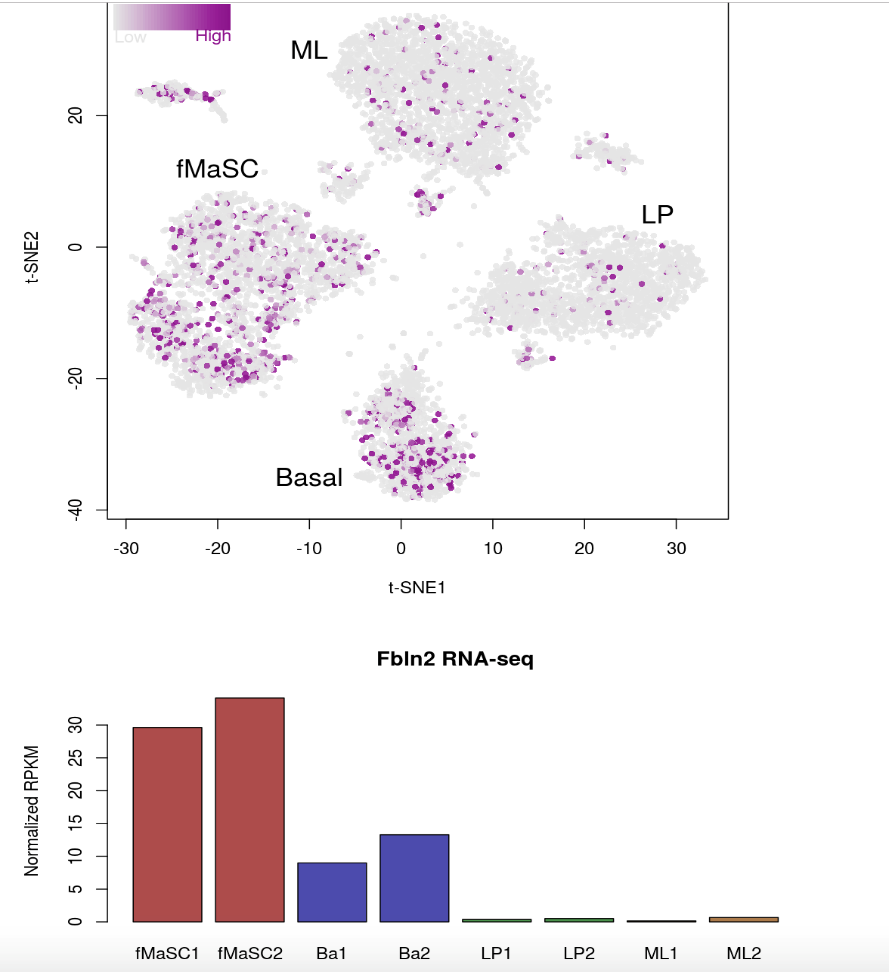


**Supplementary Figure 6.** Data visualization of the preferential expression of FBLN2 in stem cells (fMaSC1 and fMaSC2) and basal cells (Ba1 and Ba2) compared to luminal progenitors (LP1 and LP2) and mature luminal cells (ML1 and ML2).

**Supplementary Figure 7**

**
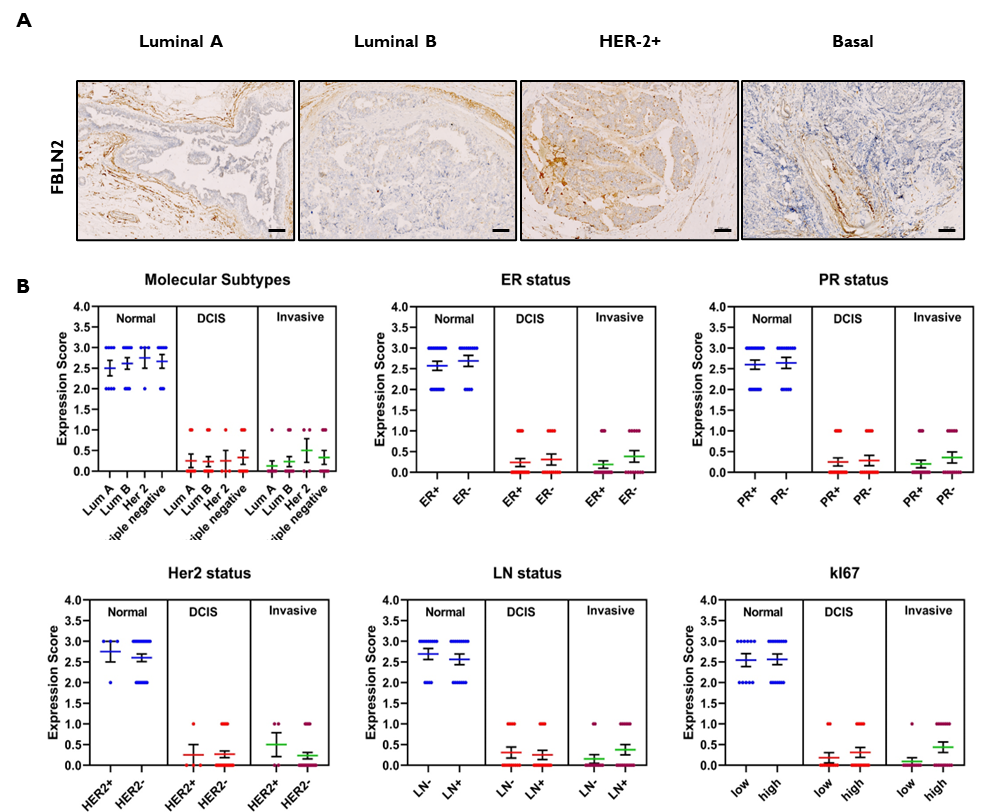
**

**Supplementary Figure 7. Immunohistochemistry staining of FBLN2 in human breast cancer patients. A:** Representative immunohistochemistry staining patterns for FBLN2 in molecular subtypes of breast cancer patients (n=36). **B**: FBLN2 staining (Expression) scores grouped by molecular subtype, or ER-, PR-, Her2-, LN-, and KI67 status, respectively.

**Supplementary Figure 8**


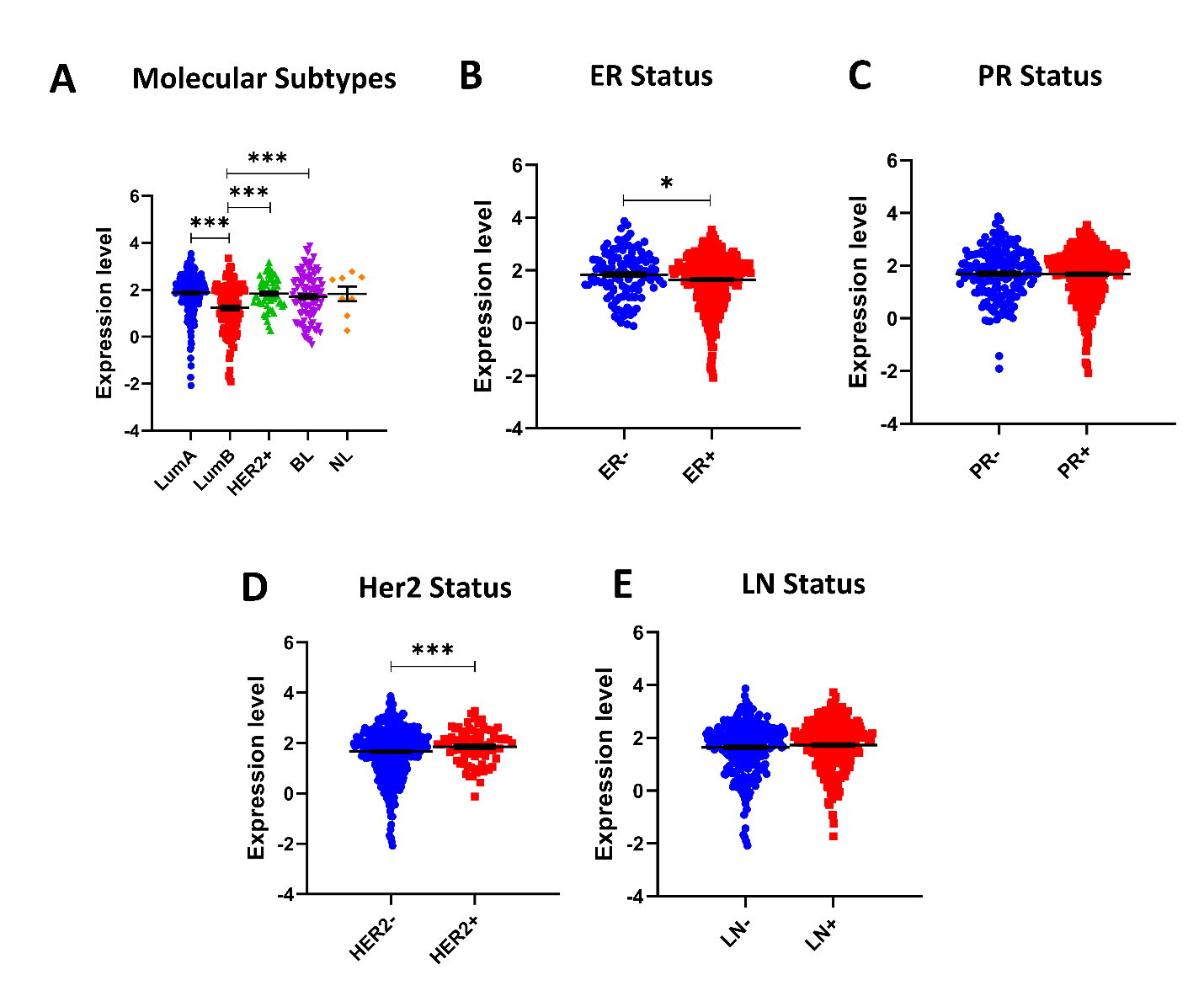


**Supplementary Figure 8.** Differential FBLN2 mRNA expression in human breast cancers based on molecular subtypes (LumA (n=230), LumB (n=125), Her2+ (n=58), Basal-like (98), and Normal like (n=8)) (A), ER status (ER+ (n=401) vs ER- (n=118)) (B), PR status (PR+ (n=340) vs PR- (n=178)) (C), Her2 status (Her2+ (n=75) vs Her2- (n=431)) (D), and LN status (LN- (n=258) vs LN+ (n=267). *P < 0.05 AND *** P < 0.001.

**Supplementary Figure 9**

**
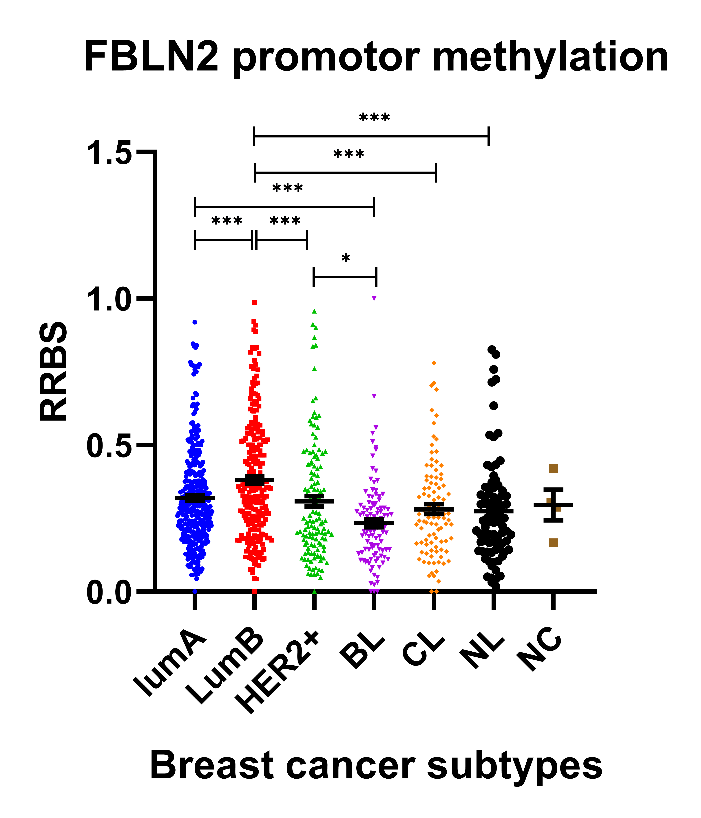
**

**Supplementary Figure 9.** FBLN2 promotor methylation profiles across breast molecular subtypes in the METBRIC dataset. LumA (n=374), LumB (n=246), Her2+ (n=133), Basal-Like (BL) (n=104), Claudin-Low (CL) (n=98), Normal-Like (NL) (n=86), and Non-Cancerous (NC) (n=4). *P < 0.05 AND *** P < 0.001.

**Supplementary Figure 10**
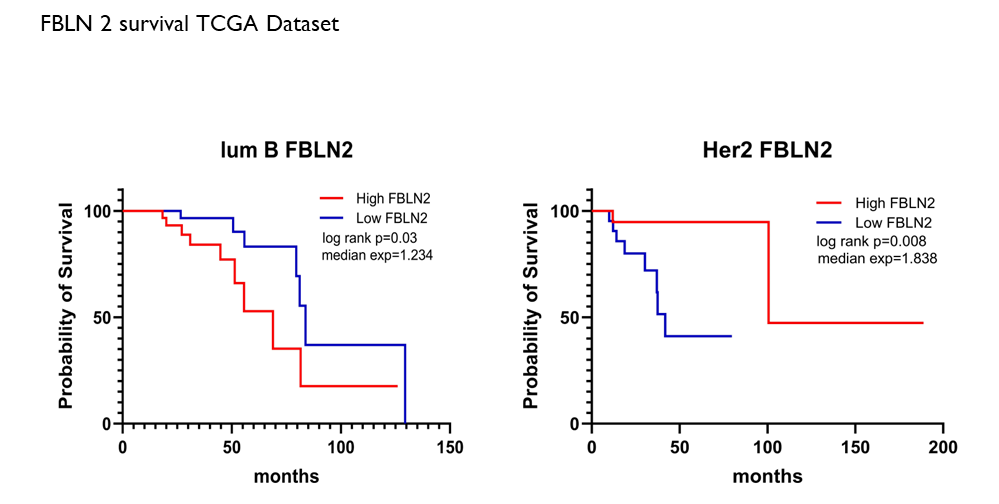


**Supplementary Figure 10.** Survival analysis of FBLN2 expression in TCGA dataset showing a better prognosis association with higher FBLN2 expression in the LumB subtype (n=117) and low FBLN2 expression associated with better prognosis in the Her2+ subtype (n=47).

**Supplementary Figure 10**


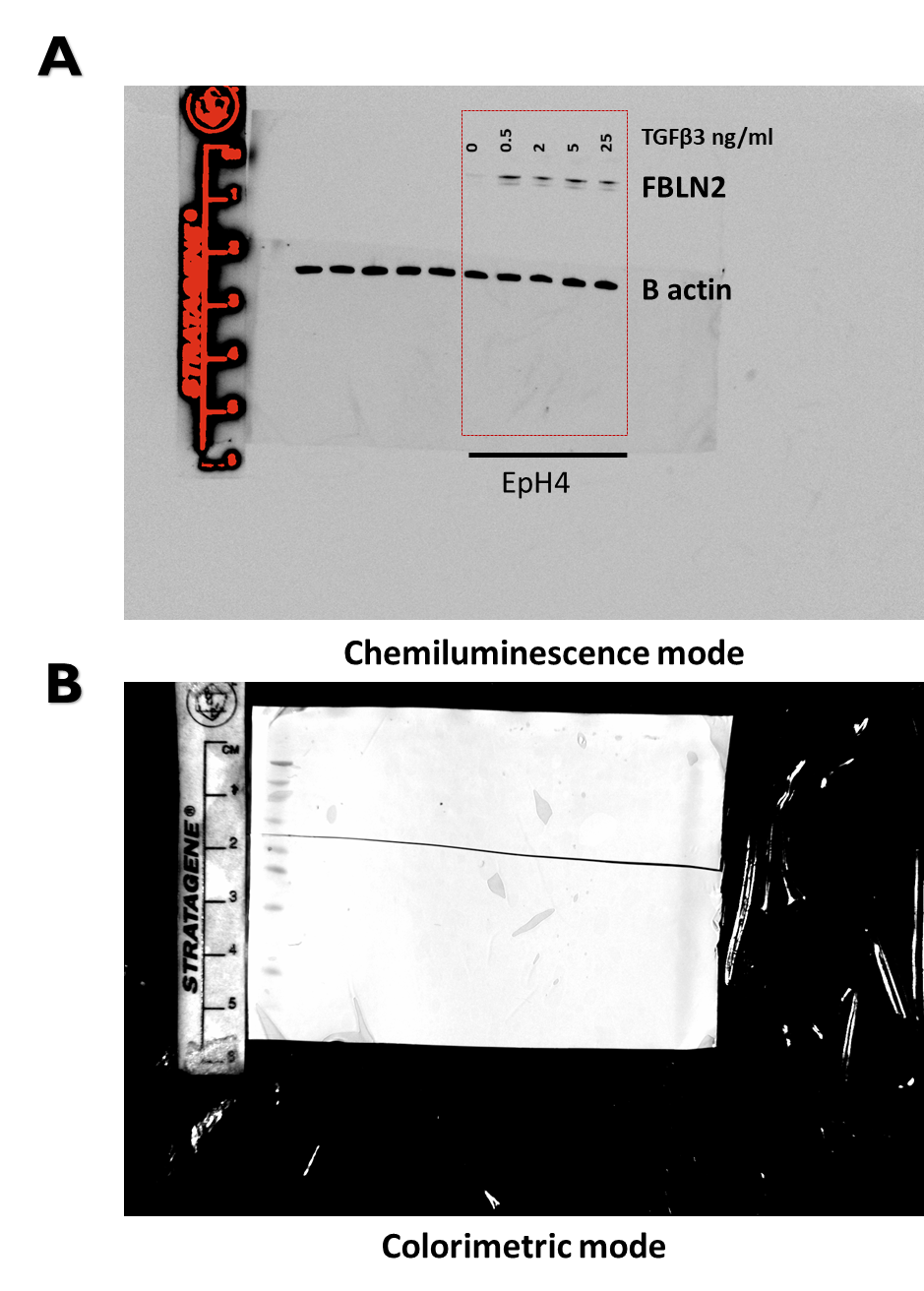


**Supplementary Figure 10. Full western blots for FBLN2 upon TGFβ3 treatment with original markings**. (**A**) Full scanned western blot showing FBLN2 and βactin. the membrane was cut at 60 KDa, the upper section was probed for FBLN2, and the lower section was probed for βactin. (**B**) The colorimetric image shows where the membrane was originally cut

**Supplementary Figure 11**


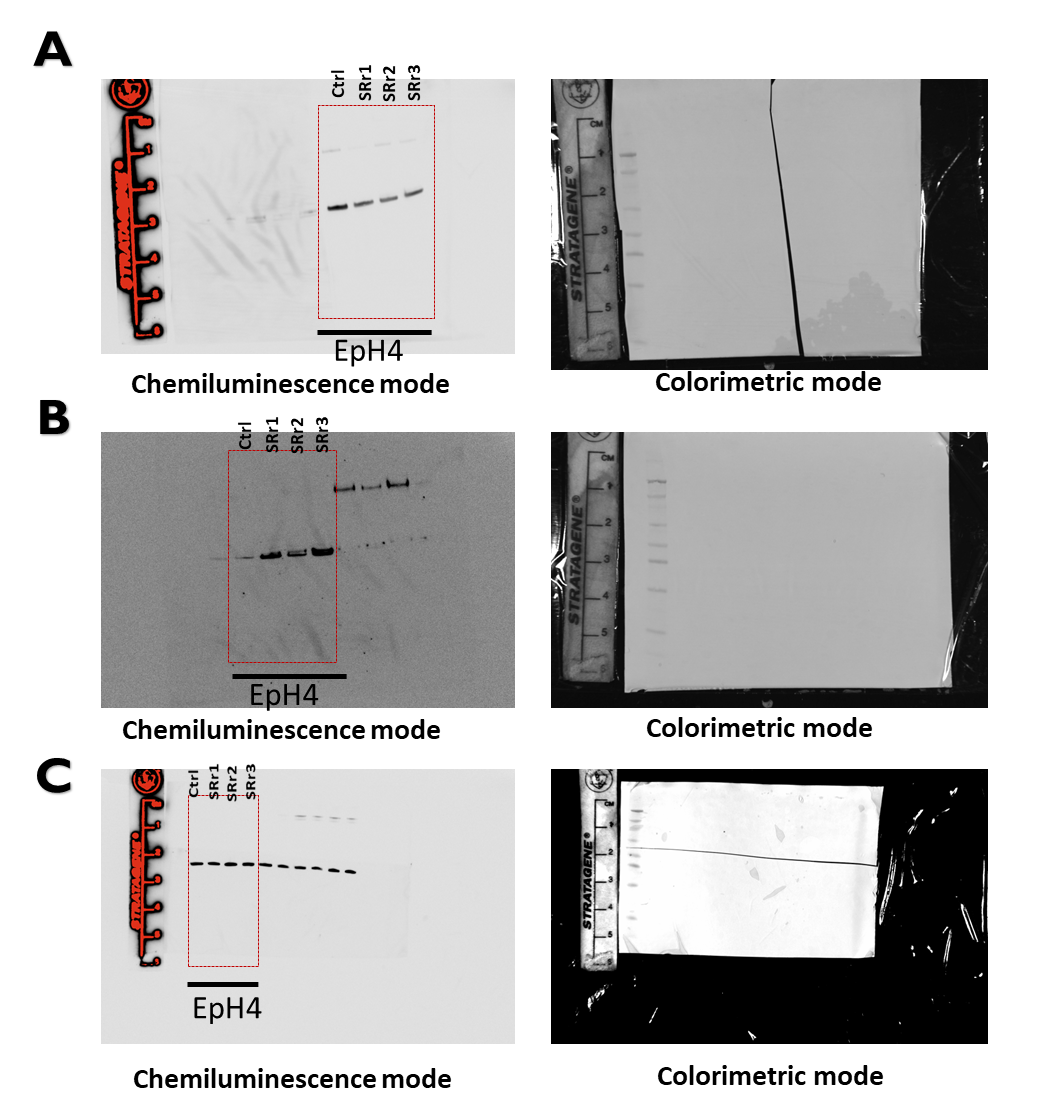


**Supplementary Figure 11. Full western blots for Krt14, Krt18, and βactin with original markings.** (**A**) Full scanned western blot showing Krt14 chemiluminescence (left) and colorimetric mode (right) (**B**) Full scanned western blot showing Krt18 chemiluminescence (left) and the colorimetric image shows where the membrane was originally cut (right) (**c**) Full scanned western blot showing βactin chemiluminescence (left) and the colorimetric image shows where the membrane was originally cut (right).

**Supplementary Figure 12**


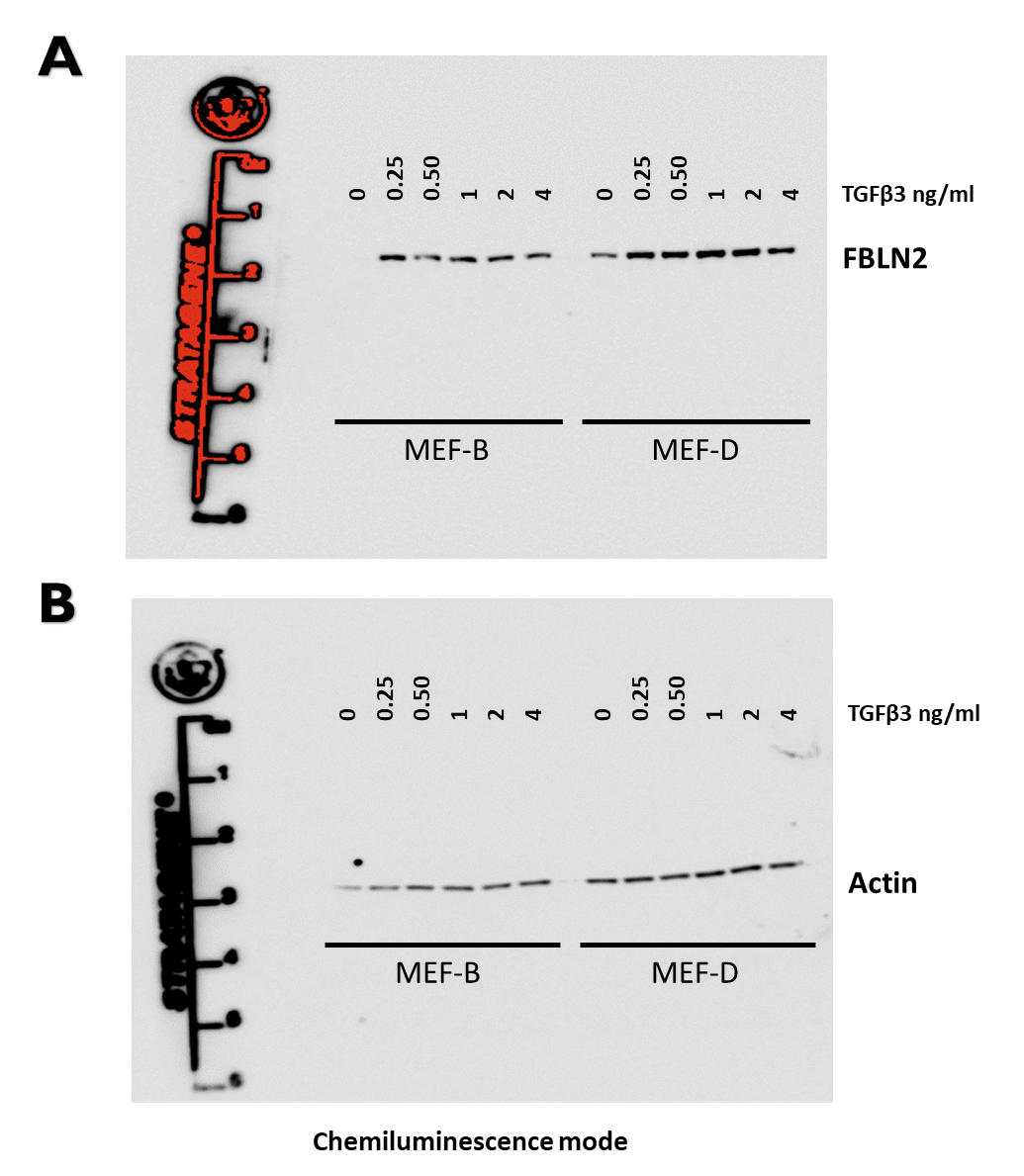


**Supplementary Figure *12*. Full western blots for FBLN2 upon TGFβ3 treatment with original markings. (A**) Full scanned western blot showing FBLN2 (**B**) Full scanned western blot showing βactin.
